# The Evolution of Combinatorial Gene Regulation in Fungi

**DOI:** 10.1371/journal.pbio.0060038

**Published:** 2008-02-26

**Authors:** Brian B Tuch, David J Galgoczy, Aaron D Hernday, Hao Li, Alexander D Johnson

**Affiliations:** 1 Department of Biochemistry and Biophysics, University of California, San Francisco, California, United States of America; 2 Department of Microbiology and Immunology, University of California, San Francisco, California, United States of America; University of Bath, United Kingdom

## Abstract

It is widely suspected that gene regulatory networks are highly plastic. The rapid turnover of transcription factor binding sites has been predicted on theoretical grounds and has been experimentally demonstrated in closely related species. We combined experimental approaches with comparative genomics to focus on the role of combinatorial control in the evolution of a large transcriptional circuit in the fungal lineage. Our study centers on Mcm1, a transcriptional regulator that, in combination with five cofactors, binds roughly 4% of the genes in Saccharomyces cerevisiae and regulates processes ranging from the cell-cycle to mating. In Kluyveromyces lactis and Candida albicans, two other hemiascomycetes, we find that the Mcm1 combinatorial circuits are substantially different. This massive rewiring of the Mcm1 circuitry has involved both substantial gain and loss of targets in ancient combinatorial circuits as well as the formation of new combinatorial interactions. We have dissected the gains and losses on the global level into subsets of functionally and temporally related changes. One particularly dramatic change is the acquisition of Mcm1 binding sites in close proximity to Rap1 binding sites at 70 ribosomal protein genes in the K. lactis lineage. Another intriguing and very recent gain occurs in the C. albicans lineage, where Mcm1 is found to bind in combination with the regulator Wor1 at many genes that function in processes associated with adaptation to the human host, including the white-opaque epigenetic switch. The large turnover of Mcm1 binding sites and the evolution of new Mcm1–cofactor interactions illuminate in sharp detail the rapid evolution of combinatorial transcription networks.

## Author Summary

In explaining the diversity of organisms on Earth, it is increasingly evident that evolutionary changes in when and where genes are expressed provide a crucial source of variation. By using genome-wide transcription factor localization experiments in S. cerevisiae, *K. lactis*, and *C. albicans*, combined with comparative genomics across many more yeast species, we examined how a large combinatorial transcription circuit evolves over the course of hundreds of millions of years. Combinatorial regulation is pervasive in eukaryotic organisms and is thought to allow for increased specificity and integration of multiple signals in the control of gene expression. Our studies focused on one prolific combinatorial regulator, Mcm1, which, in combination with five cofactors, binds and regulates genes functioning in a diverse range of cellular processes in S. cerevisiae. We found evidence of massive network rewiring, including high rates of gain and loss of Mcm1 binding sites and the formation of new Mcm1–cofactor combinations and the breaking of old ones. We propose that the multiple protein–protein and protein–DNA interactions that specify transcription in combinatorial circuits allow for a richness of compensatory mutations and thereby provide ample opportunity for both adaptive and neutral evolution.

## Abstract 3

Experimental approaches combined with comparative genomics show how a large combinatorial transcription circuit has rewired over evolutionary time scales.

## Introduction

The recent genome sequencing and annotation of the major model organisms established that organismal complexity does not scale in a simple way with gene count. This discordance is consistent with earlier proposals that “tinkering” with gene regulation may be a particularly powerful mode of evolution [1–3]. In principle, changes in when and where, and thereby in what combinations, genes are expressed can help to explain changes in organismal complexity over longer time scales. Over shorter time scales, the contributions of changes in gene regulation to phenotypic variation have been clearly demonstrated [4,5]. For example, small changes in gene regulation underlie the gain and loss of wing spots in *Drosophila* species [6] and armor in stickleback fish [7].

The plasticity of gene regulatory networks is of interest because it presumably relates directly to the ability of these networks to generate phenotypic novelty [8]. The potential for rapid turnover (gains and losses) of transcription factor binding sites was predicted on theoretical grounds [9–11] and was supported by comparisons of *cis* regulatory sequence both within and between species [12–15]. Recently, experimental localization of four transcription factors across the mouse and human genomes revealed that binding sites have diverged appreciably between these two species [16]. Analogous experiments performed on two transcription factors from closely related yeasts led to similar conclusions [17], although in this case, it was not clear how the differences in binding related to gains and losses of *cis*-acting sequences.

The ascomycete lineage, which includes the model yeast S. cerevisiae, serves as a powerful framework for investigating the general impact of regulatory evolution, because several of its members are particularly easy to study experimentally. These include the model yeast S. cerevisiae, the dairy yeast K. lactis, and the human pathogen C. albicans. S. cerevisiae and K. lactis diverged more recently than either did from C. albicans; the divergence of S. cerevisiae and C. albicans is thought to have occurred on the order of 300 million years ago [18]. To date, only a handful of comparative gene regulation studies have been carried out in fungi. These include a few large-scale analyses of changes in gene expression [19] and *cis* regulatory motifs [20,21] as well as some smaller-scale studies [22–24] focusing on sets of co-regulated genes. Whereas the whole-network studies have generally uncovered an abundance of divergence, the smaller-scale studies have characterized this divergence in greater detail or provided mechanistic insight into transcriptional rewiring.

Here we take an approach intermediate in scale and attempt to characterize the evolution of a large combinatorial circuit comprised of the MADS-box transcriptional regulator Mcm1 and each of its cofactors. Mcm1 has been intensively studied in S. cerevisiae and, in most cases, it is found as a homodimer that binds DNA cooperatively with other sequence-specific DNA binding cofactors to regulate sets of genes, which we refer to here as regulons. Five regulons have been identified in S. cerevisiae where Mcm1 acts in combination with a second transcriptional regulator. Mcm1 joins with the following: (1) MATα2 to turn off the **a**-specific genes (**a**sgs), (2) MATα1 to turn on the α-specific genes (αsgs), (3) Fkh2 and Ndd1 to activate G2/M-specific genes, (4) Yox1 to repress the M/G1-specific genes, and (5) Arg80 and Arg81 to either repress or activate the arginine metabolic genes [25]. Because Mcm1 itself is not generally regulated, it is typically the regulation of its cofactors that produces the effect of differential gene regulation at each of the Mcm1–cofactor regulons [25]. For example, it is the regulated binding of Mcm1′s cofactor Yox1 that leads to the M/G1-specific expression of genes in the Mcm1-Yox1 regulon [26]. At these Mcm1–cofactor regulons, Mcm1 is thought to increase specificity through added protein–DNA and protein–protein interactions [27].

Previously we showed Mcm1 to be at the center of a rewiring event that led to the replacement of one cofactor (MATa2) with another (MATα2) [24]. In principle, the free energy gain contributed by the interaction between Mcm1 and its flanking cofactor could catalyze evolutionary change by expanding the space of *cis*-regulatory sequences that yield appropriate gene regulation. For instance, mutations that strengthen Mcm1′s interaction with its cofactor or with DNA can compensate for mutations to the cofactor–DNA interaction, thereby expanding the possibilities for cross-reaction with a new DNA binding protein. This idea bears at least a formal similarity to the neutral networks in RNA sequence space studied by Fontana and colleagues [28]. Because Mcm1 participates in many combinatorial interactions in S. cerevisiae, and because it regulates a large number of genes, we felt that Mcm1 provided a particularly strong entry point to study the evolution of combinatorial networks.

To study this problem we performed ChIP-Chip (chromatin immunoprecipitation, analyzed genome-wide using microarrays) on Mcm1 in three species (S. cerevisiae, *K. lactis*, and C. albicans) and combined this data with informatics analyses across 32 fungal species. We found that all five Mcm1–cofactor regulons currently characterized in S. cerevisiae are present at least in limited form in K. lactis and C. albicans, suggesting an ancient origin of these regulons. Although the Mcm1–cofactor interaction is typically conserved and a small set of core target genes remains part of the regulon in each species, most of these regulons have undergone substantial divergence through gain and loss of *cis*-acting sequences. On the global level, substantial gain and loss of Mcm1 binding sites is also evident. Although some of this, as discussed above, is due to target genes moving in and out of existing regulons, much of it is due to the evolution of entirely new Mcm1–cofactor regulons. We highlight two specific instances in which combinatorial regulation by Mcm1 and a cofactor is gained; in one case, we observe the large-scale convergent evolution of regulation at the ribosomal genes and in the other, we describe a very recent gain of regulation that was likely shaped by the selective pressures of the human host. The picture that emerges from this study is one of massive transcriptional rewiring in species that span approximately the same range of protein sequence divergence as human, fish, and sea squirt [29,30]. This rewiring consists of both rapid turnover of *cis*-acting sequence and the formation of new combinations of regulatory proteins.

## Results

### Mcm1 Binds Upstream of Approximately 4% of Genes in S. cerevisiae and Approximately 12% of Genes in K. lactis and C. albicans

Mcm1 was chromatin immunoprecipitated (ChIP-ed) from S. cerevisiae, K. lactis, and *C. albicans* **a** cells using peptide antibodies custom designed for the Mcm1 ortholog of each species. To maximize the detection of Mcm1 binding, each strain was grown under two different conditions known to stimulate binding of Mcm1: yeast extract peptone dextrose (YEPD) medium and pheromone-inducing medium with α pheromone (details in the Text S1). Immunoprecipitate and whole-cell extract samples were competitively hybridized to custom-designed Agilent microarrays that tile 60mer probes at a median spacing of 66, 59, and 79 base pairs (bp) across the genomes of S. cerevisiae, K. lactis, and C. albicans, respectively (Figure S1). For each species/condition, the ChIP-Chip was performed twice and the two biological replicates were combined in downstream data processing. Data were processed by a variety of methods, and it was determined empirically that the Joint Binding Deconvolution (JBD) algorithm [31] provides the best combination of consistency across species and accuracy on a test set of previously characterized S. cerevisiae binding sites (see Text S1). Complete ChIP profiles for all experiments can be viewed at: http://genome.ucsf.edu/mcm1_evolution/.

**Figure 1:**
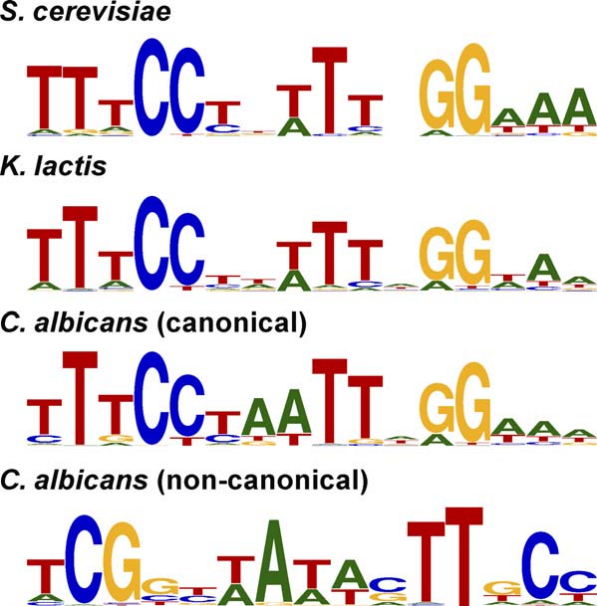
Mcm1 *cis-*Regulatory Motifs in Three Species The four *cis-*regulatory motifs identified by searching a high-confidence set of Mcm1-bound regions in the indicated species. In C. albicans, a noncanonical motif was found in addition to the canonical Mcm1 motif.

The majority of regions that JBD called as bound by Mcm1 contained at least one instance of the well-characterized Mcm1 binding motif [32,33]. We therefore decided to incorporate motif information into our final criteria for Mcm1-bound segments. De novo motif finding by MEME [34] on a set of high-confidence bound regions from JBD yields Mcm1 binding site motifs that are roughly the same in each species (Figure 1); the motif deduced de novo from S. cerevisiae closely resembles previously described Mcm1 recognition sequences. In C. albicans, there was a large subset of bound regions without a canonical Mcm1 motif. These regions are largely explained by the appearance of a noncanonical motif (Figure 1), discussed later.

Parameter cutoffs for JBD statistics and the motif *p*-value were chosen that correctly call 85% (28 of 33 genes) of our S. cerevisiae test set as positives while also calling an additional 219 of 5,769 genes as bound. Details regarding test set selection are provided in the Text S1 along with a discussion of false-positive rates and receiver operator characteristic plots (Figure S8) evaluating a variety of parameter value choices. These same cutoffs used for the S. cerevisiae data yield 626 of 5,327 genes bound in K. lactis and 761 of 6,090 genes bound in C. albicans (gene lists in Table S1). For these and all subsequent calculations, Mcm1 targets from the two growth conditions examined have been pooled.

### Genes Bound in Any One Species Are only Moderately Likely To Be Regulated in One of the Other Two Species

After defining Mcm1 targets in each species, we sought to evaluate the overlap of these targets between species. We mapped orthologs using an existing algorithm [24], which was modified to reduce directional bias (Text S1, section titled: “Mapping orthologous gene sets”), on an updated database of open reading frame (ORF) sequences from 32 fully sequenced genomes (Table S2).

**Figure 2:**
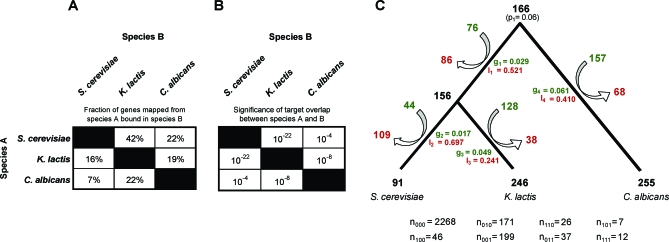
Comparison of Mcm1-Bound Target Genes in Three Species (A and B) Mcm1 targeted gene sets are compared in a pairwise fashion between species. (A) The number of genes mapped from species A and also found to be in the Mcm1 bound gene set of species B, as a fraction of the total genes bound in species A that can be mapped to species B. (B) The significance (hypergeometric *p*-value) of each pairwise overlap. (C) The inference of gain and loss rates (green and red, respectively) along each branch of the rooted three species phylogeny. The inferred number of genes added and removed from the Mcm1 regulon is listed at the top and bottom of an arrow flanking each branch. The total counts for each of the eight possible occurrence patterns used as input to the inference algorithm are presented below the tree.

Genes bound by Mcm1 in each species A were then “mapped to” one of the other two species B via our ortholog map. The number of genes “mapped from” A and also found to be in the Mcm1 bound gene set of B was counted and is displayed as a fraction of the total genes bound in species A that can be mapped to species B (Figure 2A). Note the lack of symmetry; comparing the Mcm1 bound gene set of species A to that of B is not the same as comparing the bound gene set of species B to that of A because of the different total number of Mcm1-bound genes in the different species. Overlap *p*-values were also calculated for each species pair by using the hypergeometric distribution (Figure 2B).

There is significant overlap in Mcm1-targeted genes between each pair of species (*p* < 10^−3^). As might be expected, conservation is strongest between the two more closely related species, S. cerevisiae and K. lactis, with 42% of mapped S. cerevisiae Mcm1 targets also bound by K. lactis Mcm1. However, as the lower frequency (16%) of K. lactis Mcm1-mapped targets bound by S. cerevisiae Mcm1 indicates, the K. lactis Mcm1 target set is much larger. Interestingly, the C. albicans Mcm1 target set overlaps more significantly with the K. lactis Mcm1 set than it does with the S. cerevisiae Mcm1 set, indicating that a sizeable fraction of the extra genes bound by K. lactis Mcm1 are shared with C. albicans Mcm1 and are therefore likely to have been lost as Mcm1 targets on the branch leading to S. cerevisiae (see next section).

For simplicity, we have focused here on only those genes that can be mapped in a 1:1 fashion between species. However, similar results are obtained when genes with more complex interspecies mappings (e.g., 2:1) are included. To rule out the possibility that our results were biased by the exact parameters chosen, we repeated the analysis with a variety of parameter choices and obtained similar results (Figure S9).

### Mcm1 Binding Site Turnover Is Extensive, with Sizeable Gain and Loss Rates

To assess the prevalence of gain and loss of Mcm1 binding sites across the three-species phylogeny, we constructed a model with nine parameters: four gain rates and four loss rates, corresponding to each of the four branches of the rooted tree, and a single parameter representing the probability of an Mcm1 binding site at the root of the tree (Figure 2C). We take as our dataset the Mcm1-binding occurrence patterns at each of the 2,766 genes that can be mapped between S. cerevisiae, K. lactis, and C. albicans in a 1:1:1 fashion via our ortholog mapping. There are eight such patterns, e.g., the pattern “101” for hypothetical gene X indicates an Mcm1 binding site is present upstream of gene X in S. cerevisiae and C. albicans, but not in K. lactis. We devised a modified maximum-likelihood algorithm to infer the gain and loss rates on each branch of the three-species phylogeny. A more thorough description of this procedure is given in the Text S1.

The results show a high degree of binding site turnover on all branches of the tree. For example, we estimate that the last common ancestor of S. cerevisiae and K. lactis had Mcm1 binding sites at 156 genes. Since divergence, Mcm1 binding sites were gained at 44 genes and lost at 109 genes in the S. cerevisiae lineage. Likewise, Mcm1 binding sites were gained at 128 genes and lost at 38 genes in the K. lactis lineage. Thus, present day S. cerevisiae and K. lactis have only 38 Mcm1-targeted genes in common. We do not believe that this analysis is biased by any systematic failures to detect Mcm1 binding sites in our ChIP experiments—either through experimental biases or because the growth conditions chosen did not promote Mcm1 binding. In cases where Mcm1 is bound upstream of a gene in one species but not in the other two species, the Mcm1 motif is generally not present in those other two species as well (Text S1, section titled: “Mcm1 DNA motifs are not present at genes that are not bound”). In the sections that follow, we will further dissect the changes in combinatorial regulation that give rise to the conservation and divergence summarized in Figure 2C.

### There Is a Small Conserved Core of “Ancestral Mcm1-Bound Genes”

**Figure 3:**
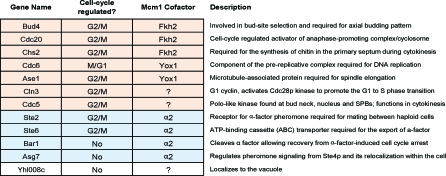
The Ancestral Mcm1-Bound Genes These twelve genes are targets of Mcm1 in all three species. For each gene, the cell-cycle phase of increased expression [65] (if applicable), the relevant Mcm1 cofactor (if known), and a brief functional annotation is listed. Cell-cycle– and mating-type–regulated genes are shaded orange and blue, respectively.

If we consider just the subset of genes that has a 1:1:1 mapping in our ortholog table, only 12 genes (∼13% of the genes bound in S. cerevisiae) are part of the Mcm1 circuit in all three species (Figure 3). If gene duplications are allowed, the number of genes in S. cerevisiae with at least one “ortholog” bound in K. lactis and C. albicans is 45 (∼18% of the genes bound in S. cerevisiae). Presumably this conserved set of target genes reflects a conserved role played by Mcm1 in the common ancestor as well as in the three modern species.

The set of ancestral Mcm1-bound genes is clearly enriched for genes regulated by the cell cycle (Figure 3, shaded orange) and mating type (Figure 3, shaded blue). The latter is confirmation of results from our previous study [24] describing the conservation of membership within the **a**-specific gene regulon despite the dramatic switch from positive regulation by MATa2 to negative regulation by MATα2. In S. cerevisiae, the cell cycle genes listed are regulated by the Mcm1 cofactors Fkh2/Ndd1 and Yox1. The conservation of these genes as targets of Mcm1 prompted us to inquire whether combinatorial control by Mcm1 and each of its S. cerevisiae cofactors was also conserved since the time when S. cerevisiae, K. lactis, and C. albicans diverged from a common ancestor.

### Most Mcm1–Cofactor Interactions Observed in Modern S. cerevisiae Emerged Early, but Their Target Genes Have Changed Dramatically

**Figure 4:**
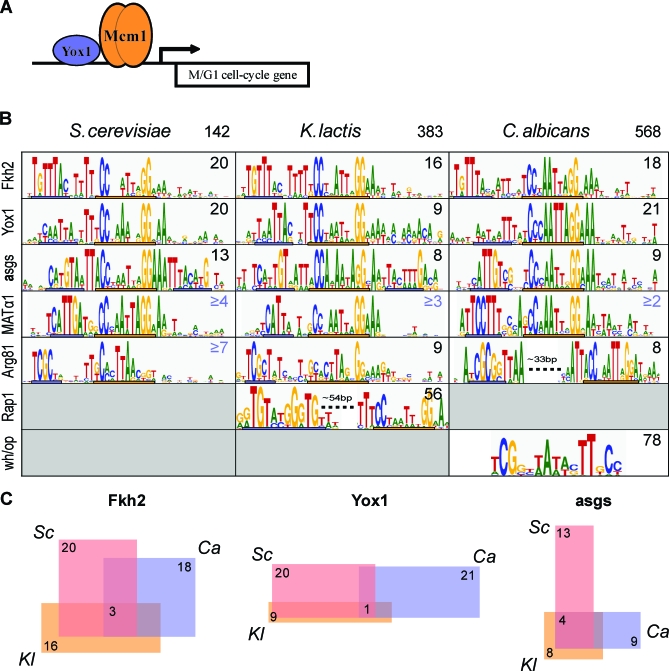
Comparison of Mcm1–Cofactor Regulons across Species (A) An example schematic of the Mcm1 homodimer and its cofactor, Yox1, binding in close proximity upstream of an M/G1-specific cell cycle gene. (B) Mcm1 associated *cis-*regulatory motifs discovered across the three species in this work. Each row of the table specifies an Mcm1–cofactor regulon and each column a species. The total number of Mcm1-bound regions in each species is listed in the header row. The number of Mcm1 bound regions assigned to each Mcm1–cofactor regulon in each species is listed in the upper right corner of each cell of the table; numbers colored black are based on Mcm1 ChIP data, whereas those in blue are not and are therefore more tentative. Mcm1 binds or is predicted to bind the consensus sequence denoted by the orange bar in each cell. The known or predicted cofactor motif is denoted by a blue bar in each cell. Motif graphics were generated with WebLogo [66]. (C) The three-way overlap of target genes in the Fkh2-Mcm1, Yox1-Mcm1, and **a**sg (Mcm1-a2 or Mcm1-α2) regulons in the three species (Sc = S. cerevisiae, Kl = K. lactis, and Ca = C. albicans).

The Mcm1–cofactor regulons of S. cerevisiae were mapped to Mcm1-bound regions in K. lactis or C. albicans, and motif finding was performed to identify *cis*-regulatory elements controlling the orthologous regulons (details presented in Text S1). The results of this analysis (Figure 4A and 4B) demonstrate that most known Mcm1–cofactor interactions from S. cerevisiae are present in K. lactis and C. albicans and are therefore likely of ancient origin. In the description that follows, we first compare the *cis-*regulatory motifs of the more closely related S. cerevisiae and K. lactis and then compare these to the motifs of the more divergent C. albicans. Here we use the term “interaction” to refer to both demonstrated protein–protein interactions as well as those inferred from the co-occurrence in *cis* of two or more regulatory motifs. One caveat of this approach is that co-occurrence of motifs can arise from cooperative as well as competitive binding of two transcription factors. However, we think the latter is unlikely for most cases documented in this work, because the spacing of the motifs tends to be highly constrained and nonoverlapping, a feature typically observed for cooperative binding with Mcm1.

In general the *cis-*regulatory elements of the K. lactis and S. cerevisiae Mcm1–cofactor regulons are similar, suggesting that the corresponding Mcm1–cofactor interactions have changed little since these two lineages split. Notable exceptions are the changes seen at **a**sgs discussed previously [24] and the apparent added Fkh2 specificity flanking several of the Yox1-Mcm1 sites in K. lactis (Figure 4B). Although the latter is seen in at least a few genes in S. cerevisiae [26], this Yox1-Mcm1-Fkh2 architecture appears much more prominent in K. lactis.

Comparison of K. lactis and S. cerevisiae to the more divergent C. albicans reveals that a number of changes to *cis-*regulatory motifs have occurred over longer time scales. At the Fkh2-Mcm1 regulon, there is a shift in the placement of the Fkh2 site relative to the Mcm1 site by 1 base pair, which occurs across the entire regulon. We note that species with the tighter Fkh2-Mcm1 spacing have clear orthologs to Ndd1, a protein which in S. cerevisiae binds the Fkh2-Mcm1 complex periodically, thereby driving G2/M-specific expression [35], while those with the lengthened spacing do not. It is not known how the Fkh2-Mcm1 complex of C. albicans would function to drive G2/M-specific gene expression without an Ndd1 ortholog, although this altered spacing may provide a clue. It is also noteworthy that Fkh2 is related to another protein, Fkh1, which is derived from the yeast whole-genome duplication event [36], meaning that these two genes found in S. cerevisiae map to a single gene in K. lactis and C. albicans. It is known that Fkh2 binds DNA cooperatively with Mcm1, but that Fkh1 does not [37]. Given the evidence for the Fkh2-Mcm1 motif in K. lactis and C. albicans, we infer that this interaction is ancestral to the species under study and that after duplication, only Fkh2 retained the ability to bind cooperatively with Mcm1.

The *cis*-regulatory motif at the MATα1-Mcm1 regulon has clearly changed as well, indicating that MATα1, despite its obvious conservation, recognizes distinct DNA motifs in different species. However, the altered MATα1 motif observed in C. albicans is not necessarily incompatible with the S. cerevisiae protein, a surmise based on previous mutagenesis studies [38]. Despite this change in motif, experimental evidence indicates that MATα1′s function as an activator of αsgs is the same in S. cerevisiae and C. albicans [39].

Once it was determined that most Mcm1–cofactor pairings are conserved across the species we examined, we then determined to what extent the set of genes in their corresponding regulons was also conserved. The motif matrices for each of the Mcm1–cofactor pairs were used to score the entire set of Mcm1-bound sequences in each species and thus to define the members of each Mcm1–cofactor regulon in each species (Text S1). We found that the number of targets in each regulon is roughly the same across the three species, but the precise set of members is not. However, within each regulon, there is a small, core set of conserved genes (Figure 4C). For example, the Fkh2-Mcm1 regulon consists of roughly 20 genes in each species, but only three genes are part of the regulon in all three species. Previously we showed that for the **a**sg regulon at least, this core is conserved throughout the yeasts spanning the lineage of S. cerevisiae and C. albicans [24]. A similar promoter sequence analysis with the Mcm1-Fkh2 matrices supports a conserved core within this regulon as well (unpublished data). For example, the promoters of BUD4 and CDC20 have strong matches to the Fkh2-Mcm1 matrix in most species within the lineage spanning S. cerevisiae and C. albicans. Thus, turnover within these regulons is not a purely stochastic process, but rather is constrained in some respects by purifying selection.

### Lineage-Specific Gain and Loss of Mcm1–Cofactor Interactions Is also Evident

As summarized in Figure 2C, this study revealed many specific instances of gains and losses of Mcm1 regulation across the ascomycete lineage. The large number of changes seen at the global level, however, can not be fully accounted for by binding site turnover within the ancestral Mcm1–cofactor regulons alone (Figure 4C). In the following section, we highlight three examples of large-scale rewiring events, chosen for their particular clarity and their relevance to well-developed systems.

#### Mcm1 and Rap1 binding sites at ribosomal genes in K. lactis.

There are 378 genes bound by Mcm1 in K. lactis, but not in S. cerevisiae or C. albicans. Fifty-nine of these are annotated as constituents of the cytosolic ribosome in S. cerevisiae (*p* < 10^−45^). In total, 70 of the 101 genes annotated as cytosolic ribosomal genes are bound by Mcm1 in K. lactis. A closer examination reveals that the 70 ribosomal genes bound by Mcm1 encode for structural constituents of the small or large subunits, whereas the other 31 genes tend to encode for translational accessory proteins such as the acetyltransferase Nat5 and the mRNA decapping factor Pat1.

Of the three species we studied, only K. lactis has Mcm1 sites at its ribosomal genes, therefore we examined a broader range of fungi to determine with greater resolution whether this pattern likely results from gains in the K. lactis lineage or losses in the S. cerevisiae and C. albicans lineages. To do so, we mapped the 162 cytosolic ribosomal genes (Gene Ontology [GO] identification number 0005830) from S. cerevisiae to 31 other fully sequenced fungal genomes and then performed de novo motif finding on the promoters of these genes (500-bp upstream of the translational start) with MEME [34].

**Figure 5:**
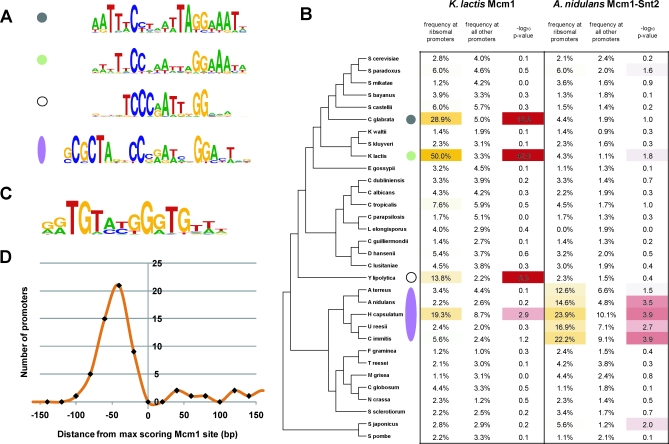
Evolution of Mcm1 Binding Sites at Ribosomal Genes in the Ascomycete Lineage (A and B) Convergent evolution of Mcm1 motifs at ribosomal genes. (A) Four Mcm1-like *cis*-regulatory motifs discovered in a MEME search of the ribosomal gene promoters of 32 fully-sequenced ascomycete genomes. The motifs were discovered in the species indicated by the colored circles and oval in (B). The Mcm1-like motif of the A. nidulans branch has a tandem cofactor motif that is nearly identical to that derived from Snt2 ChIP-Chip experiments in S. cerevisiae [43]; we therefore predict that the Snt2 orthologs of the A. nidulans lineage are the Mcm1 cofactors at the ribosomal genes of this lineage. (B) The Mcm1 motifs from K. lactis (green circle) and the A. nidulans lineage (lavender oval) were used to score ribosomal promoters across the ascomycete lineage and thus to verify that presence of the Mcm1 motifs is limited to the four lineages in which Mcm1-like motifs were found de novo by MEME. The significance of motif enrichment at the ribosomal promoters of each species was determined by comparison to genome-wide background frequencies of occurrence using the binomial distribution. See Text S1 for description of the ascomycete phylogeny reconstruction [24,67]. (C) An additional motif similar to that recognized by Rap1 in S. cerevisiae was discovered in the MEME search of K. lactis ribosomal promoters. (D) In K. lactis, the positioning of Rap1-like motif instances is constrained relative to Mcm1 motif instances.

To our surprise, motifs resembling that of Mcm1 were found at ribosomal genes in several species—C. glabrata, *K. lactis*, and *Yarrowia lipolytica*—which do not cluster phylogentically. Furthermore, a motif resembling Mcm1, plus an unknown cofactor, was found in the branch spanning Aspergillus nidulans to *Histoplasma capsulatum* (Figure 5A). To verify that the presence of the Mcm1-like motifs at ribosomal genes was limited to just C. glabrata, *K. lactis*, Y. lipolytica, and the A. nidulans branch, we scored the ribosomal gene promoters (1 kb upstream of the translational start) of each species with the Mcm1 motif matrices (Figure 5B). Indeed, evidence for Mcm1-like motifs at ribosomal genes is limited to just the aforementioned species.

Formally, we can not rule out the possibility that Mcm1 may bind indirectly to ribosomal gene promoters in species in which we have not performed ChIP. However the changes in *cis-*acting sequence are striking and imply, at the very least, a change in mechanism. We also cannot formally rule out the possibility that a smaller than statistically significant fraction of the ribosomal genes is regulated by Mcm1 in some other species. However, given that the ribosome plays such an essential role in the cell and that even small reductions in the expression of a single ribosomal gene relative to the others can lead to substantial slowing of growth rate [40], the latter seems unlikely as well.

If we suppose that the loss of established Mcm1 regulation of the ribosomal genes is just as costly as gaining Mcm1 regulation of ribosomal genes, then the evolution of Mcm1 at ribosomal genes is most parsimoniously explained by four independent gains. The next most parsimonious scenario is three gains and two losses. If we posit a single gain of regulation, then at least five losses must occur as well.

Our discovery of Mcm1 at the ribosomal genes in K. lactis (but absent from the orthologous genes of S. cerevisiae and C. albicans) prompted us to search for a possible cofactor. The same MEME search that identified the Mcm1 motif at the ribosomal gene promoters of K. lactis also identified a *cis-*regulatory motif that is similar in sequence to that recognized by Rap1 in S. cerevisiae [41] (Figure 5C). Indeed, it was shown previously that the Rap1-like motif is present at ribosomal gene promoters in S. cerevisiae, K. lactis, and closely related yeasts, and thus it was inferred that this motif was present at ribosomal genes in the last common ancestor of S. cerevisiae and K. lactis [21]. By searching the cytosolic ribosomal gene promoters of K. lactis for the presence of maximal scoring Mcm1 and Rap1 motifs (log_10_-odds scores > 2.0), we find that the newly discovered Mcm1 sites are semi-strictly positioned at a median 54-bp downstream (with respect to the ORF) of Rap1 sites (Figure 5D). Although the distance constraint is not as strict as those typically seen for other Mcm1 cofactors (Figure 4B), we believe it is likely that Rap1 is a newly discovered Mcm1 cofactor in K. lactis. To summarize, it seems likely that in the K. lactis lineage Mcm1 binding sites were gained at 70 ribosomal genes and that a combinatorial interaction between Mcm1 and a pre-existing ribosomal regulator, Rap1, was formed.

#### Mcm1, Arg80, and Arg81 binding sites at arginine metabolic genes.

One of the more prominent aspects of the loss-gain diagram of Figure 2C is the relatively higher rate of loss on the branch leading to S. cerevisiae. This finding is consistent with the results of the pairwise comparison, which suggested the existence of a set of genes conserved between K. lactis and C. albicans, but lost on the branch to S. cerevisiae. The set of genes with an Mcm1 binding site in K. lactis and C. albicans, but lacking sites in S. cerevisiae, totals 58 (in S. cerevisiae) and is enriched for arginine metabolic genes (GO identification 0006525; *n* = 5; *p* < 10^−6^).

**Figure 6:**
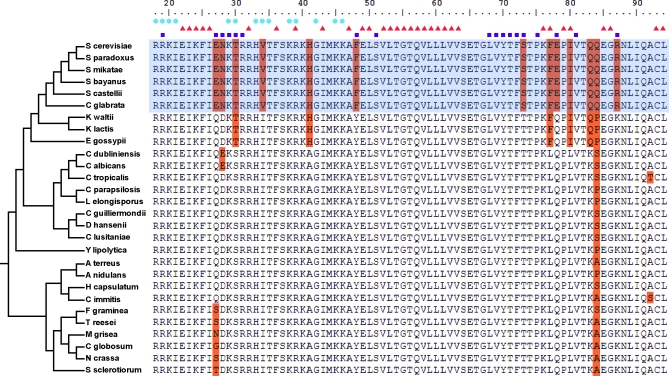
Substitutions within the MADS Box Domain of Mcm1 There are a few substitutions to the MADS box domain of Mcm1 (orange) against a background of strong conservation (white) within the hemiascomycete and euascomycete lineages. The shaded box indicates Mcm1 orthologs from species that also have an Mcm1 duplicate (named Arg80 in S. cerevisiae). Mcm1 residues forming contacts with α2, Mcm1, or DNA in the crystal structure of the α2-Mcm1-DNA ternary complex (Protein Databank ID: 1mnm) are indicated above the alignment with squares, triangles, and circles, respectively. Note the strong correlation between those species having substitutions at the α2 interacting residues, those species with an Mcm1 duplicate, and those species thought to be using a purely negative mode of **a**sg regulation by Mcm1 and α2[24].

Mcm1 has a duplicate in S. cerevisiae, Arg80, which arose after the divergence of K. lactis and S. cerevisiae. Our observations are most consistent with a model whereby Mcm1′s ancestral role, collaborating with the Mcm1 cofactor Arg81 in arginine metabolism, was, at least in part, handed off to its duplicate Arg80. Although previous in vitro work demonstrated that Mcm1 and Arg80 form heterodimers at operator sequences found upstream of arginine metabolic genes in S. cerevisiae [42], our Mcm1 ChIPs and the Mcm1 and Arg80 ChIPs performed by others [43] suggest that in vivo, these dimers might more typically consist of two molecules of Arg80. Based on our identification of Mcm1 binding at arginine metabolic genes in K. lactis and C. albicans (Figure 4B), Mcm1′s role interacting with Arg81 at arginine genes is inferred to be ancient, having evolved prior to the divergence of S. cerevisiae and C. albicans. The timing of the handoff to Arg80 is coincident with not only the whole-genome duplication, but also with the switch from a putatively hybrid (positive and negative) mode of **a**sg regulation by MATa2 and MATα2 to a purely negative mode by MATα2, and with roughly 50% of all substitutions in the DNA binding domain of Mcm1 (see alignment in Figure 6). It is plausible that this handoff of some arginine regulon function to an Mcm1 duplicate “freed up” the surface of Mcm1, allowing for the strengthening of an interaction between Mcm1 and MATα2 [24].

#### Mcm1 and Wor1 binding sites at white-opaque genes in the C. albicans lineage.

As mentioned previously, the Mcm1 bound sequences of C. albicans contain a second “noncanonical” *cis*-regulatory motif (Figure 1) that strongly correlates with Mcm1 occupancy at roughly 127 genes that lack a strong match to the canonical Mcm1 motif (log_10_-odds noncanonical motif score > 4.0 and log_10_-odds canonical motif score < 3.0). To rule out possible cross-hybridization of our Mcm1 antibody to another DNA-binding protein, we repeated the ChIP of Mcm1 in C. albicans (1) in the same strain with an antibody raised against a peptide from the N terminus of Mcm1 (rather than the C terminus as before) and (2) in a *myc*-tagged Mcm1 strain[44] using an antibody to the *myc*-epitope. Both ChIPs were hybridized to C. albicans tiling arrays (normalized to whole cell–extract DNA), and both results validate the enrichment of Mcm1 seen at the noncanonical motif (unpublished data). Furthermore, at the promoters of these genes, the noncanonical motif tends to be centered with respect to the peak of Mcm1 ChIP enrichment (unpublished data), suggesting either direct binding of Mcm1 to this motif or tight interaction of Mcm1 with another transcriptional regulator that recognizes this motif. The noncanonical motif is absent from the Mcm1 bound regions of S. cerevisiae and K. lactis, and the noncanonical Mcm1-bound genes of C. albicans are generally not bound by Mcm1 in either S. cerevisiae and K. lactis.

Among the 110 Mcm1-bound C. albicans genes with very strong noncanonical motif scores (log_10_-odds > 4.5) are several genes annotated with functions in cell adhesion (*n* = 9; GO identification 0007155; *p* < 10^−5^), biofilm formation (*n* = 7; GO identification 0042710; *p* < 10^−5^) and regulation of white-opaque switching (Wor1/orf19.4884, Efg1/orf19.610, and Wor2/orf19.5992) [45,46]. These three processes are important for C. albicans to interact with its mammalian host.

**Figure 7:**
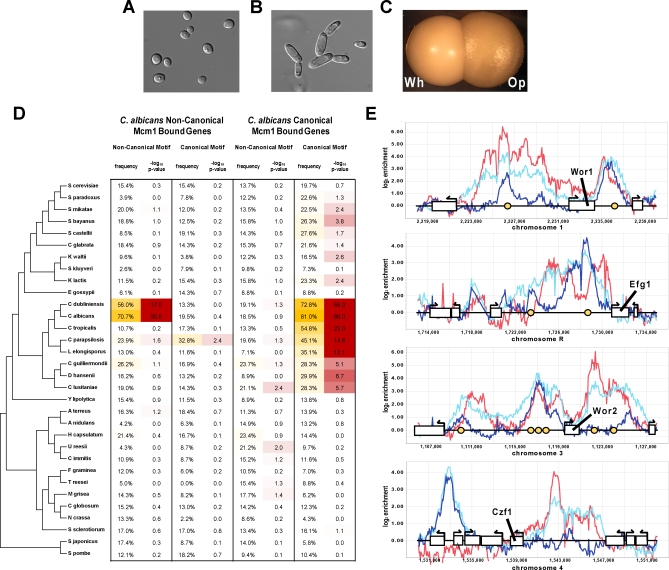
Recent Evolution of Noncanonical Mcm1 Binding Sites at White-Opaque Genes (A–C) C. albicans cell types. (A) White cells. (B) Opaque cells. (C) A white colony (Wh) and an opaque colony (Op). (D) The noncanonical and canonical Mcm1 motif matrices of C. albicans (Figure 1) were used to score promoters for two sets of genes (genes where Mcm1 is found at the noncanonical motif in C. albicans and genes where Mcm1 is found at the canonical motif in C. albicans) across the ascomycete lineage. The significance of motif enrichment at the two mapped gene sets of each species was determined by comparison to genome-wide background frequencies of occurrence using the binomial distribution. (E) ChIP-Chip profiles for Mcm1 and Wor1 in regions flanking four key regulators of the white-opaque switch [45]. Dark blue, aqua, and red lines indicate the Mcm1 ChIP of white cells, Mcm1 ChIP of opaque cells, and Wor1 ChIP of opaque cells, respectively. Yellow circles indicate a noncanonical Mcm1 motif.

To determine when Mcm1 regulation at the noncanonical binding site arose, we mapped the 110 Mcm1-bound genes with very strong noncanonical motif scores to orthologs in each of the other 31 species and scored the promoters of these ORFs (2 kb upstream of the translational start) for presence of the noncanonical Mcm1 binding motif (Figure 7D). The presence of the noncanonical Mcm1 motif at these genes is clearly limited to C. albicans and C. dubliniensis (a very closely related human pathogen), suggesting that either the noncanonical regulatory motif arose just prior to the C. albicans–C. dubliniensis split or that it evolved earlier and has just recently moved to this set of genes. That the noncanonical motif was not seen at the S. cerevisiae and K. lactis Mcm1-bound genes increases our confidence that the gain of this noncanonical regulatory motif was very recent. By way of comparison, when we mapped the genes bound by Mcm1 at the canonical motif in C. albicans to the other species, one sees clear evidence for the canonical Mcm1 motif in species of the Debaryomyces hansenii branch and (with somewhat lowered confidence) in species as far diverged as S. bayanus and K. lactis. This observation suggests that the presence of a *cis-*regulatory element in only two very closely related species is unusual, and thus further increases our confidence that the noncanonical motif is recently evolved.

The role of the noncanonical Mcm1 binding site in C. albicans white-opaque switching bears further scrutiny, as the regulatory circuit behind this epigenetic phenomenon has been studied intensively. Briefly, C. albicans forms two distinctive types of cells, white and opaque, which differ in their appearance [47] (Figure 7A–7C), the genes they express [39], their mating behavior [48] and interaction with host sub-environments [49,50]. Both states are heritably maintained for many generations, and switching between them occurs at low frequency (∼1/10^4^ cell generations). A master regulator of white-opaque switching, Wor1, has been identified [51–53] and has been shown to bind many white- and opaque-specific genes [45].

Comparison of the Mcm1 and Wor1 ChIPs in opaque cells reveals a striking overlap of Mcm1 and Wor1 binding in the upstream regions of all known critical regulators of white-opaque switching, including WOR1 itself (Figure 7E). Genome-wide, 36 of the 110 genes with noncanonical Mcm1 binding sites are also bound by Wor1 (33%; hypergeometric *p* < 10^−24^), suggesting an interaction between the two proteins. These results indicate the intimate involvement of Mcm1 and the noncanonical Mcm1 motif in white-opaque switching and raise the possibility that the evolution of this motif played an important role in the acquisition of white-opaque switching and other interactions with the host by the C. albicans lineage.

## Discussion

In this work, we have tracked the evolution of combinatorial gene regulation by the highly conserved transcriptional regulator Mcm1 and each of its known cofactors across the ascomycete fungal lineage. Our analysis shows that the genes regulated by Mcm1 have changed considerably over the evolutionary time scales represented by this lineage; our results reveal many more differences than similarities in the Mcm1 circuitry. Regulation by Mcm1 is more pervasive in K. lactis and C. albicans, where 12% of all genes are bound, than in S. cerevisiae, where 4% of genes are bound. The fraction of genes shared as targets between all three species is very low (13%–18%), and we have demonstrated that this is due to both substantial gain and loss of Mcm1 binding sites along each branch of this phylogeny (Figure 2B). The extensive amount of gain and loss observed is consistent with recent studies in mammals [16] and closely related yeasts [17] and suggests the following three possibilities: (1) there is a richness of selective advantages offered in the dynamic rewiring of gene regulatory networks, (2) there are a large number of neutral alternatives to gene regulation by Mcm1, or (3) selection on gene expression is weak. The latter possibility seems at odds with other observations such as the large fraction of genes devoted to transcriptional regulation in S. cerevisiae (∼3%), the greater-than-expected number of transcriptional regulators retained after the whole genome duplication (∼6% versus ∼3%), and the considerable conservation found in many S. cerevisiae promoters [54,55]. Additionally, the fact that many of the Mcm1 sites are enriched at functionally related genes and often found in tandem with cofactor motifs argues strongly against the hypothesis that a large number of these sites are fortuitous and nonfunctional. Gauging the relative contributions of selection versus neutral drift on the gene regulatory networks will be an exciting challenge for future research [56].

Despite the highly dynamic nature of evolution of Mcm1 regulation, we find evidence that most Mcm1–cofactor interactions characterized in S. cerevisiae are also present in K. lactis and C. albicans (Figure 4B). Although the Mcm1–cofactor pairings are conserved, the set of genes that each regulates has diverged considerably across species. Nonetheless, each Mcm1–cofactor pair targets a small core of genes conserved as part of the regulon. These regulon cores are enriched for genes functioning in the cell cycle and mating. Thus it would seem that Mcm1′s role in these processes evolved prior to the split of the species we have chosen to study. Nevertheless, even at these conserved regulons, there are many species-specific differences. For example, across an entire regulon, the spacing between Fkh2 and Mcm1 binding sites has changed in S. cerevisiae and K. lactis relative to C. albicans, as have the DNA recognition sequences of MATα1. This latter observation is particularly interesting because it suggests that the specificity of MATα1 has evolved without an accompanying gene duplication.

In addition to the conservation of Mcm1–cofactor interactions associated with cell cycle and mating, we see the evolution of new Mcm1–cofactor regulons. For example, Mcm1 binding sites are gained at the majority of ribosomal genes in K. lactis in close proximity to binding motifs for another transcription factor, Rap1 (Figure 5C and 5D). The evolution of ribosomal gene regulation has been studied previously [21], but a role for Mcm1 was not discussed. Our new results support the idea, first proposed by Tanay et al. [21], that while the protein sequence of this critical macromolecular machine has remained nearly constant, its regulation has undergone substantial diversification in yeasts. What is perhaps most surprising is our finding that the set of species that contain Mcm1 binding motifs upstream of ribosomal genes (Figure 5A and 5B; C. glabrata, *K. lactis*, *Y. lipolytica*, and the A. nidulans lineage) do not cluster phylogenetically. From this we inferred that Mcm1 binding at ribosomal genes likely evolved on four separate occasions. If further genome sequencing continues to support this result, this will serve as the largest instance of convergent regulatory evolution yet reported. The relatively sudden appearance of Mcm1 binding sites in close proximity to Rap1 sites at roughly 70 ribosomal genes in K. lactis raises another important question: Can the commonly accepted mutational processes, such as point mutation and recombination, support this scale of concomitant changes—or must some alternative mechanism for moving promoters around the genome be invoked [57,58]? One can argue that, without a redundant mechanism in place, loss or gain of Mcm1 regulation of even a single gene means losing precise control over one component of a macromolecular complex that is thought to need tight stochiometric control [40]. With further sequencing and characterization of Mcm1′s functional role at the ribosomal genes, it may become clear how such a massive regulatory change can take place at a set of genes encoding such highly conserved, tightly regulated and essential proteins.

In C. albicans, we identified the presence of Mcm1 at a noncanonical motif upstream of roughly 110 genes. The noncanonical motif differs significantly from the canonical Mcm1 motif (Figure 1), although in both cases GC-rich regions flank an AT-rich core. To our knowledge no MADS-box domain has ever been shown to bind a sequence this far diverged from the canonical Mcm1 motif. Even so, we find that noncanonical motifs tend to be centered with respect to peaks of ChIP-Chip enrichment and thus conclude that Mcm1 either binds this motif directly with some unknown cofactor or some unknown transcriptional regulator recognizes this motif and interacts strongly with Mcm1. The set of genes at which Mcm1 binds the noncanonical motif is enriched for processes such as adhesion and contains three of four known regulators of the white-opaque phenotypic switch [45]. The white-opaque switch is of considerable interest because the white and opaque states are heritable and because the two states are thought to allow adaptation to different niches within a human host [49,50]. In this vein, the evolution of regulation associated with the switch deserves special attention too, because the changes seen here represent, to our knowledge, the first gene regulatory changes to be associated with a heritable biological process and one of only a few instances implicated to play an adaptive role in fungal biology [59]. The results of our comparative analysis of 32 yeast species demonstrate that Mcm1 binding at the noncanonical motif is found only in two very closely related species, C. albicans and C. dubliniensis, and thus likely arose only very recently (Figure 7D). Moreover white-opaque switching has been described only in these two species [60], which are both pathogens of humans. Thus, the evidence so far suggests that the white-opaque switch may have arisen just prior to the divergence of *C. albicans* and C. dubliniensis and that the emergence of the noncanonical Mcm1 motif at white-opaque regulators was crucial to this development. Alternatively, the white-opaque switch may have arisen earlier, and the addition of Mcm1 regulation may have refined it in some way, affecting heritability, for example.

The picture that emerges from this study is one of massive transcriptional rewiring in species that span approximately the same range of divergence as human, fish, and sea squirt [29,30]. Mcm1 regulates hundreds of genes in S. cerevisiae, K. lactis, and C. albicans, but less than 20% of Mcm1–target gene connections are preserved in all three species. The differences arise from target genes moving in and out of ancient Mcm1–cofactor regulons, but also from the formation of new Mcm1–cofactor interactions and the loss of ancient ones. Taken together with our previous work [24], we have now provided evidence for the gain of three interactions: Mcm1 with MATα2, Mcm1 with Rap1, and Mcm1 with Wor1. We have also described loss of an interaction between Mcm1 and MATa2 and the loss of an interaction between Mcm1 and Arg81 that was preserved in an Mcm1 duplicate. In attempting to judge the relative contributions of combinatorial control per se to the evolution of transcriptional circuits, we acknowledge that the ideal “control” datasets do not exist. For example, data collected from a large noncombinatorial circuit (should one even exist) over several species would allow an objective assessment of the special contribution of combinatorial control to circuit evolution. Nonetheless, our results provide experimental and informatic support for the idea that combinatorial networks are highly evolvable [61–64], and perhaps more importantly, they document specific mechanisms by which one large combinatorial circuit has evolved.

## Methods

Detailed methods can be found in Text S1, Figure S1–S10, and Table S1–S3. The information can also be found in one complete file, Protocol S1.

## Supporting Information

Figure S1 Evaluation of Tiling Array Design Columns 1–3 contain plots for the S. cerevisiae, *K. lactis*, and C. albicans tiling array designs, respectively. See Text S1 for description.
(247 KB DOC)

Figure S2 Comparison of the Performance of ChIP Analytics (CA) and Joint Binding Deconvolution (JBD) on S. cerevisiae ChIP-Chip Data Receiver operator characteristic (ROC) plots for the three analysis methods (CA, CA_FIX, and JBD) on the ChIP-Chips of S. cerevisiae Mcm1 under two growth conditions (YEPD and α-factor). See Text S1 for further description.
(45 KB DOC)

Figure S3 Results of ChIP Analytics (CA) on the ChIP-Chip Datasets from All Three Species The enrichment *p*-value cutoff was varied (*x-*axis), and the resulting number of bound genes called is recorded, both as a fraction of all test set genes in S. cerevisiae (left *y-*axis; silver and black bars) and as a fraction of all genes in each of the three genomes (right *y*-axis; pink, purple, and blue lines).
(537 KB DOC)

Figure S4 Distributions of the Enrichment Statistic (X_bar_) ChIP Analytics X_bar_ distributions for (A) S. cerevisiae, (B) K. lactis, and (C) *C. albicans* α-factor ChIP-Chip experiments. The blue line is the ChIP Analytics (CA) Gaussian fit and the red line is our attempt at an improved Gaussian fit (CA_FIX).
(68 KB DOC)

Figure S5 Results of the Modified ChIP Analytics (CA_FIX) on the ChIP-Chip Datasets from All Three Species The modified enrichment *p*-value cutoff was varied (*x-*axis), and the resulting number of bound genes called was recorded, both as a fraction of all test set genes in S. cerevisiae (left *y-*axis; silver and black bars) and as a fraction of all genes in each of the three genomes (right *y-*axis; pink, purple, and blue lines).
(586 KB DOC)

Figure S6 Estimated Influence Functions for Each Experiment For each experiment, we estimate an influence function as the average of the relative enrichment as a function of distance from the 50 strongest, idealized peaks in each experiment. Sc = S. cerevisiae, Kl = K. lactis, and Ca = *C. albicans.*
(272 KB DOC)

Figure S7 Results of JBD on the ChIP-Chip Datasets from All Three Species The cutoffs for JBD statistics (*p*_binding_ and ∑[*p*_binding_ * *strength*_binding_]) were varied (*x-*axis), and the resulting number of bound genes called was recorded, both as a fraction of all test set genes in S. cerevisiae (left *y-*axis; silver and black bars) and as a fraction of all genes in each of the three genomes (right *y-*axis; pink, purple and blue lines).
(621 KB DOC)

Figure S8 Results of JBD Integrated with Motif Information on the ChIP-Chip Datasets from All Three Species The cutoffs for the motif p-value and the JBD statistics (∑[*p*_binding_ * *strength*_binding_] and ∑[*p*_binding_ * *strength*_binding_] for motif override) were varied (*x-*axis), and the resulting number of bound genes called was recorded, both as a fraction of all test set genes in S. cerevisiae (left *y-*axis; silver and black bars) and as a fraction of all genes in each of the three genomes (right *y-*axis; pink, purple, and blue lines). Here the cutoff for *p*_binding_ is 0.2.
(1.2 MB DOC)

Figure S9 Robustness of Pairwise Species Comparison Results to Parameter Choices Cutoffs for the four parameters that define the set of genes called as Mcm1 bound in each species were varied (shown in each blue table), and the results of the pairwise species comparison (described in detail in the Results section) were recomputed. The first 3 × 3 table in each column indicates the number of genes bound by Mcm1 in each species A that can be mapped to one of the other two species B in a 1:1 manner. The second table indicates the number of genes mapped from A and also found to be in the Mcm1 bound gene set of B, as a fraction of the total genes bound in species A that can be mapped to species B. The third table indicates the significance (hypergeometric *p*-value) of each pairwise overlap.
(144 KB DOC)

Figure S10 The Three-Branch (Star) and Four-Branch, Rooted Three Species Tree Models (93 KB DOC)

Protocol S1 Combined Supporting Document (3.8 MB DOC)

Table S1 Lists of Mcm1-Bound Genes in Each Species (19 KB TXT)

Table S2 List of Genomes Used in This Work (91 KB DOC)

Table S3 A Test Set of Mcm1-Regulated S. cerevisiae Genes (145 KB DOC)

Text S1 Supporting Methods (242 KB DOC)

## Abbreviations

αsgs: α-specific genes
**a**sgs: **a**-specific genes
ChIP: chromatin immunoprecipitation
ChIP-Chip: chromatin immunoprecipitation, analyzed genome-wide using microarrays
GO: Gene Ontology
JBD: joint binding deconvolution algorithm
MAT: mating-type
ORF: open reading frame
