# Supplementary figures and images for: The Evolution of Combinatorial Gene Regulation in Fungi
Source: PLoS Biol. 2008 Feb 26;6(2):e38. doi: 10.1371/journal.pbio.0060038 (PMC2253631; doi:10.1371/journal.pbio.0060038)

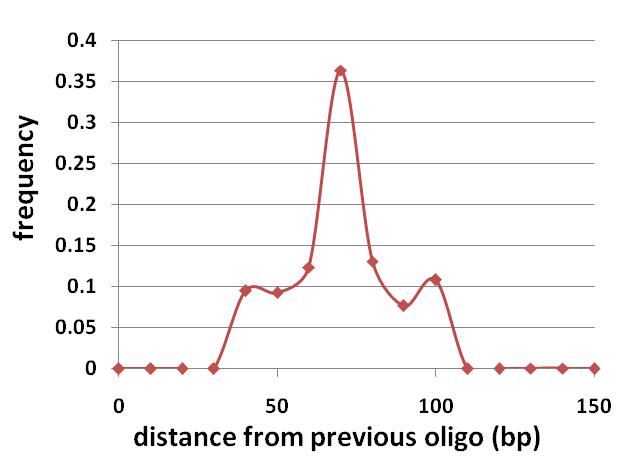

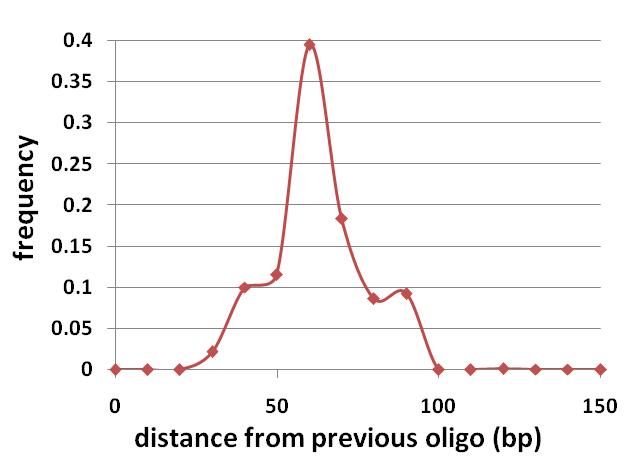

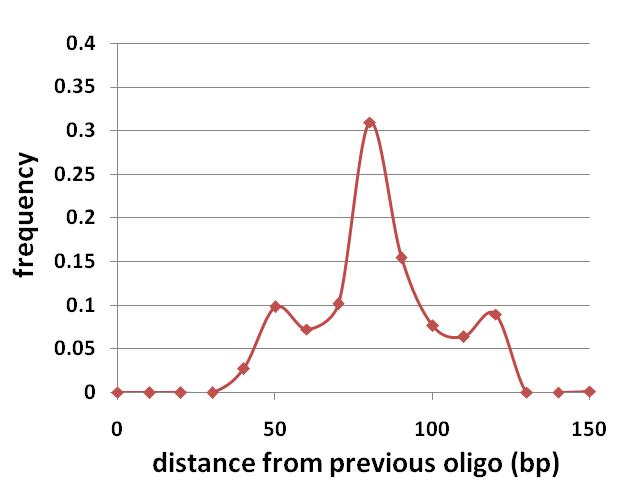


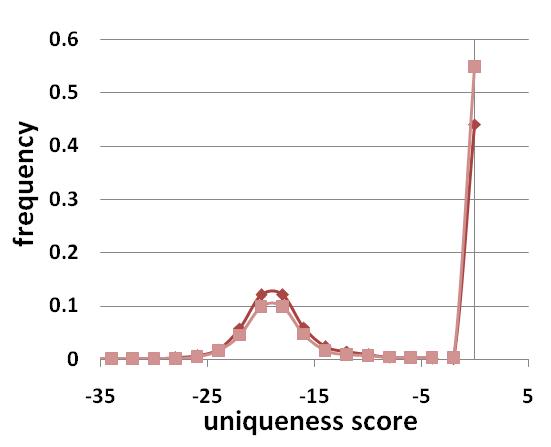

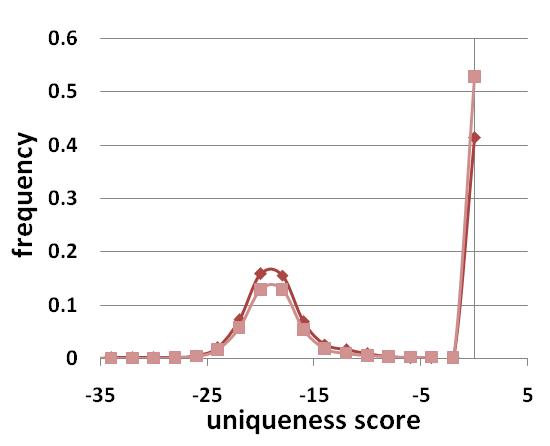

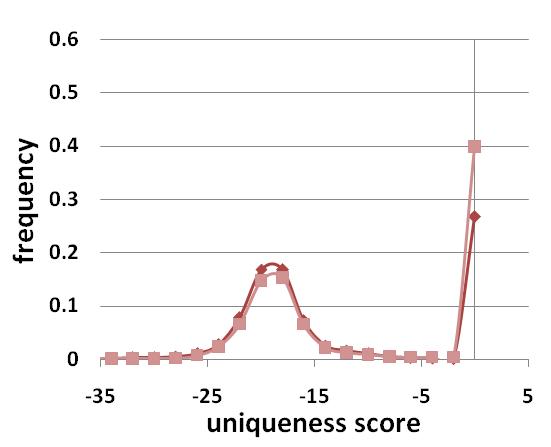


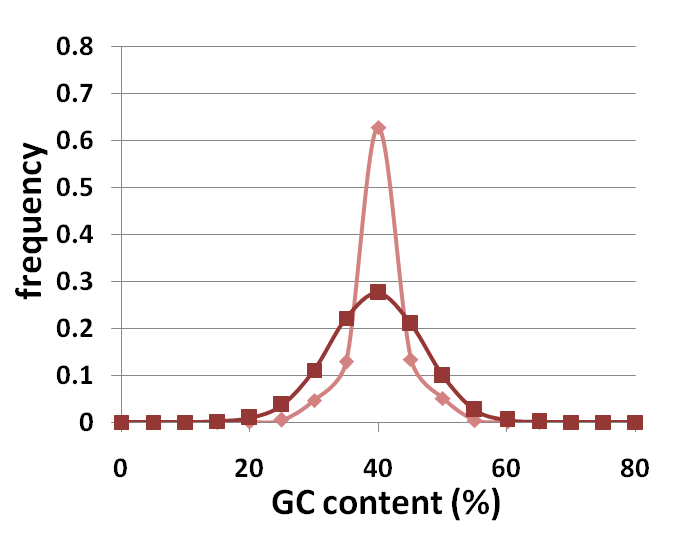

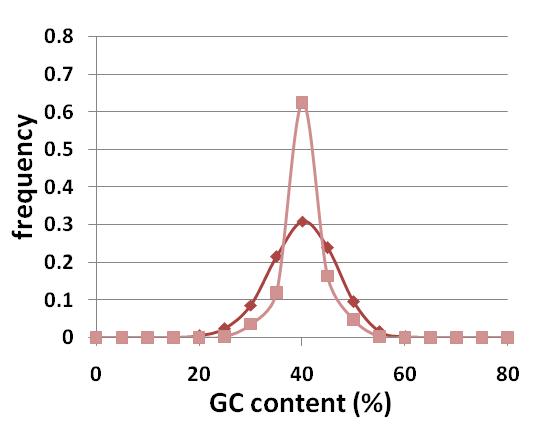

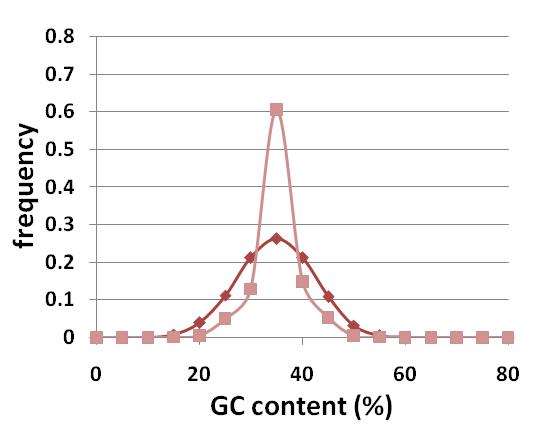

Supplement: Figure S1 — Columns 1–3 contain plots for the S. cerevisiae, K. lactis, and C. albicans tiling array designs, respectively. See Text S1 for description. (247 KB DOC) [file pbio.0060038.sg001.doc]

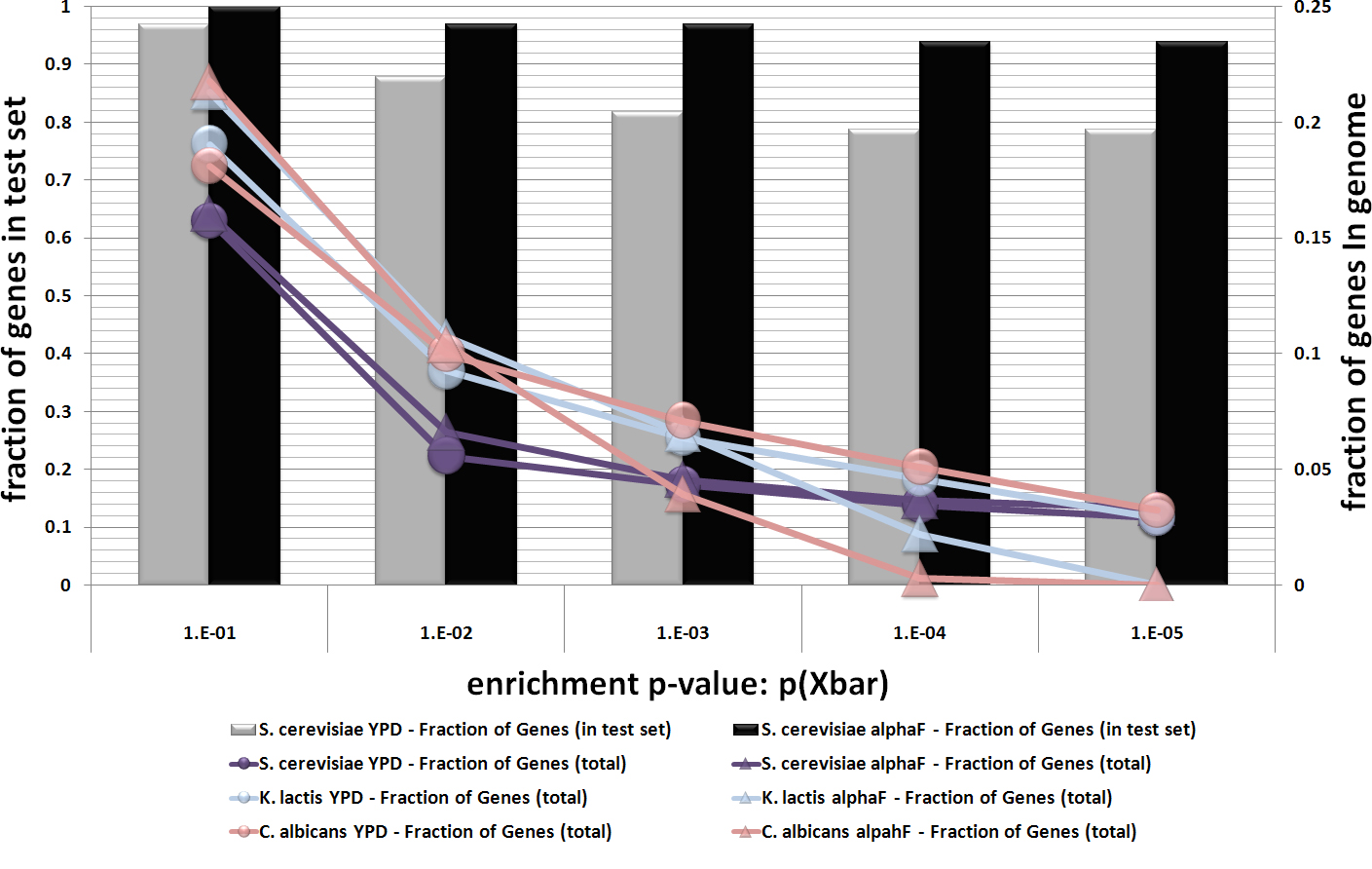

Supplement: Figure S3 — The enrichment p-value cutoff was varied (x-axis), and the resulting number of bound genes called is recorded, both as a fraction of all test set genes in S. cerevisiae (left y-axis; silver and black bars) and as a fraction of all genes in each of the three genomes (right y-axis; pink, purple, and blue lines). (537 KB DOC) [file pbio.0060038.sg003.doc]

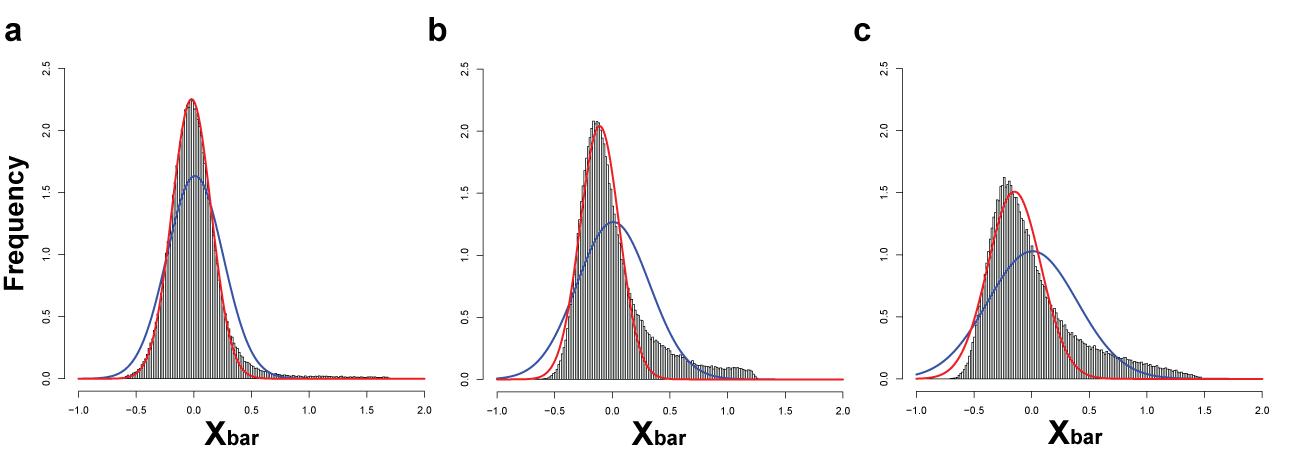

Supplement: Figure S4 — ChIP Analytics Xbar distributions for (A) S. cerevisiae, (B) K. lactis, and (C) C. albicans α-factor ChIP-Chip experiments. The blue line is the ChIP Analytics (CA) Gaussian fit and the red line is our attempt at an improved Gaussian fit (CA_FIX). (68 KB DOC) [file pbio.0060038.sg004.doc]

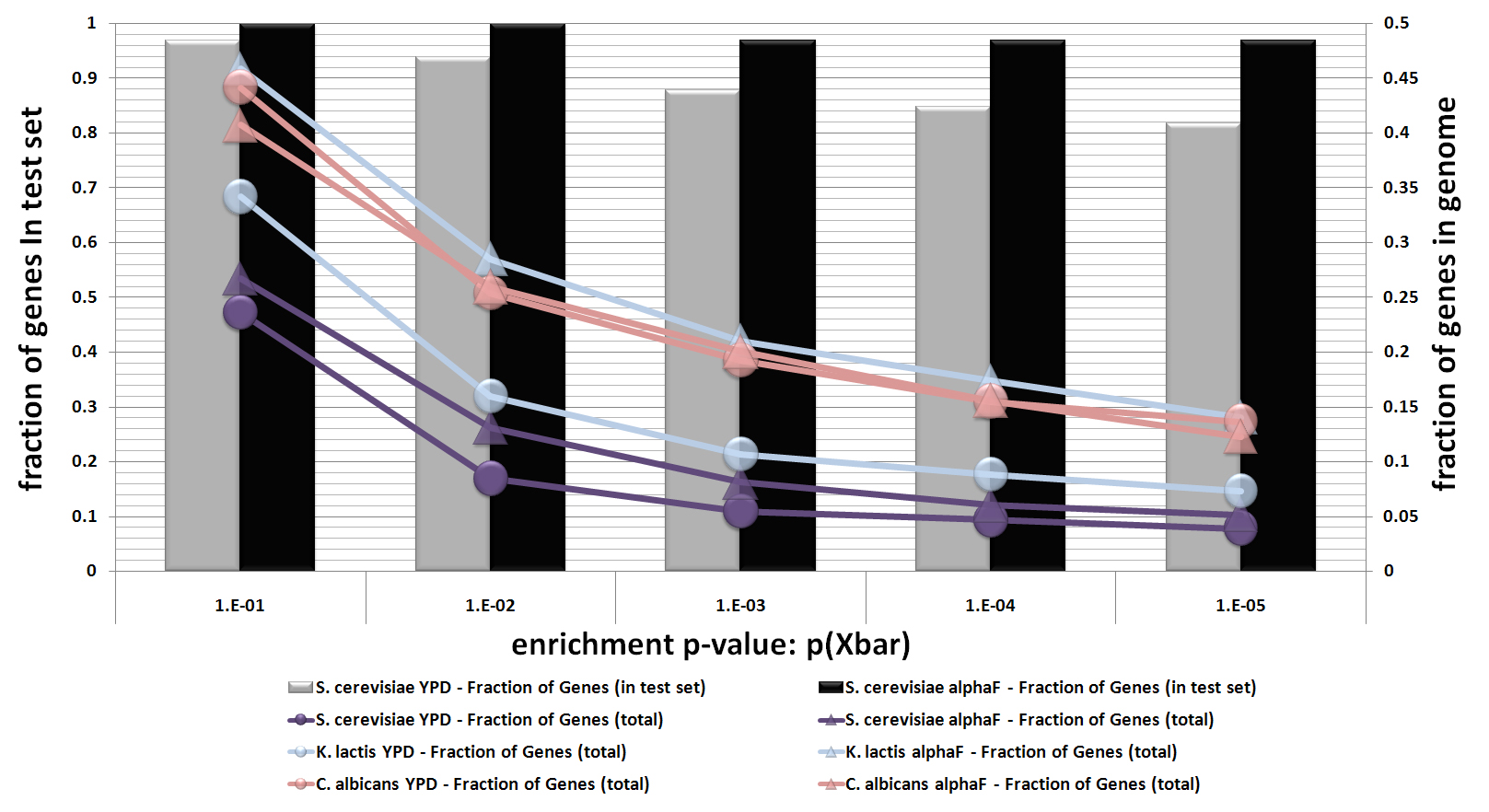

Supplement: Figure S5 — The modified enrichment p-value cutoff was varied (x-axis), and the resulting number of bound genes called was recorded, both as a fraction of all test set genes in S. cerevisiae (left y-axis; silver and black bars) and as a fraction of all genes in each of the three genomes (right y-axis; pink, purple, and blue lines). (586 KB DOC) [file pbio.0060038.sg005.doc]

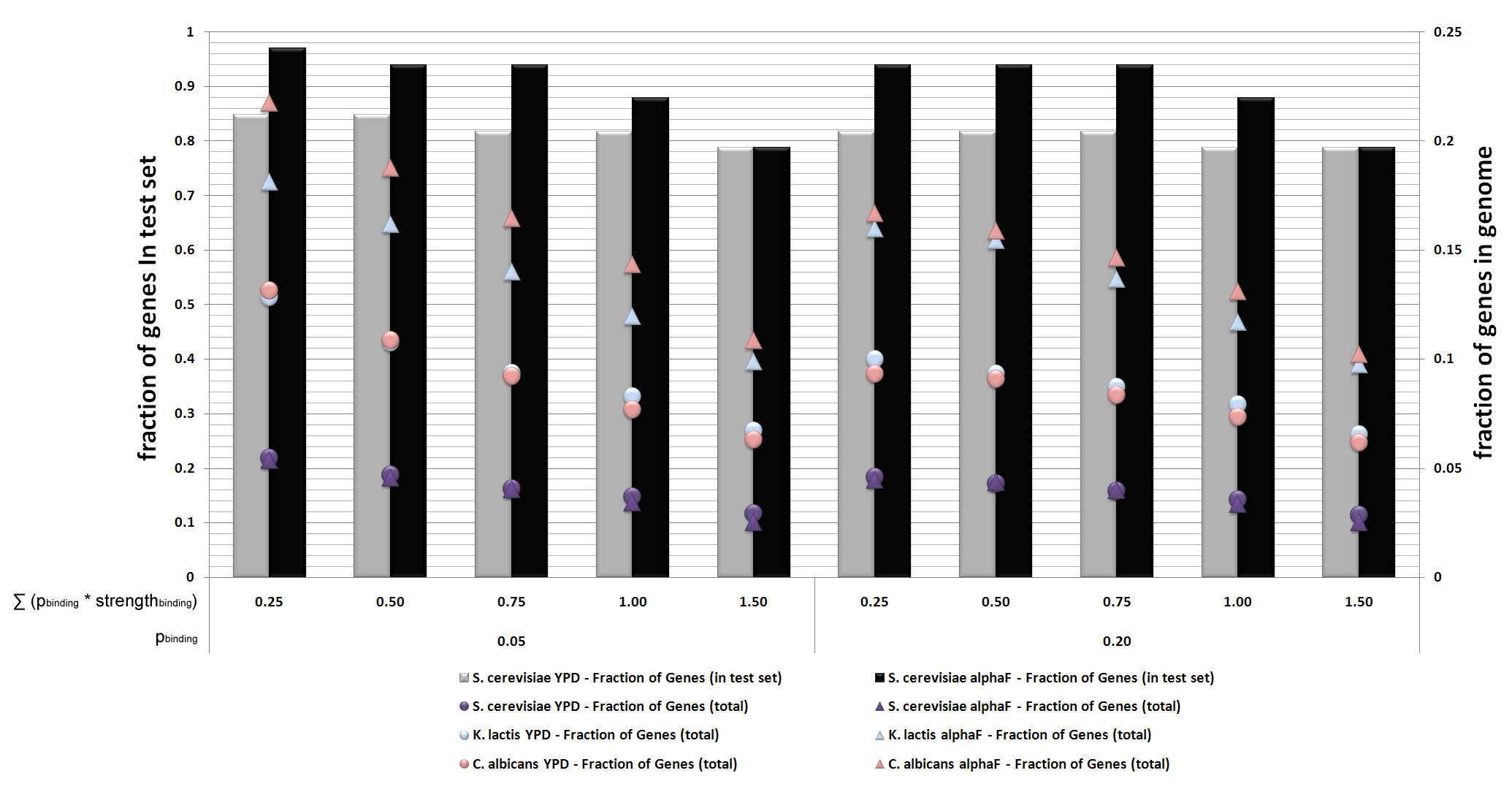

Supplement: Figure S7 — The cutoffs for JBD statistics (p binding and ∑[p binding * strength binding]) were varied (x-axis), and the resulting number of bound genes called was recorded, both as a fraction of all test set genes in S. cerevisiae (left y-axis; silver and black bars) and as a fraction of all genes in each of the three genomes (right y-axis; pink, purple and blue lines). (621 KB DOC) [file pbio.0060038.sg007.doc]

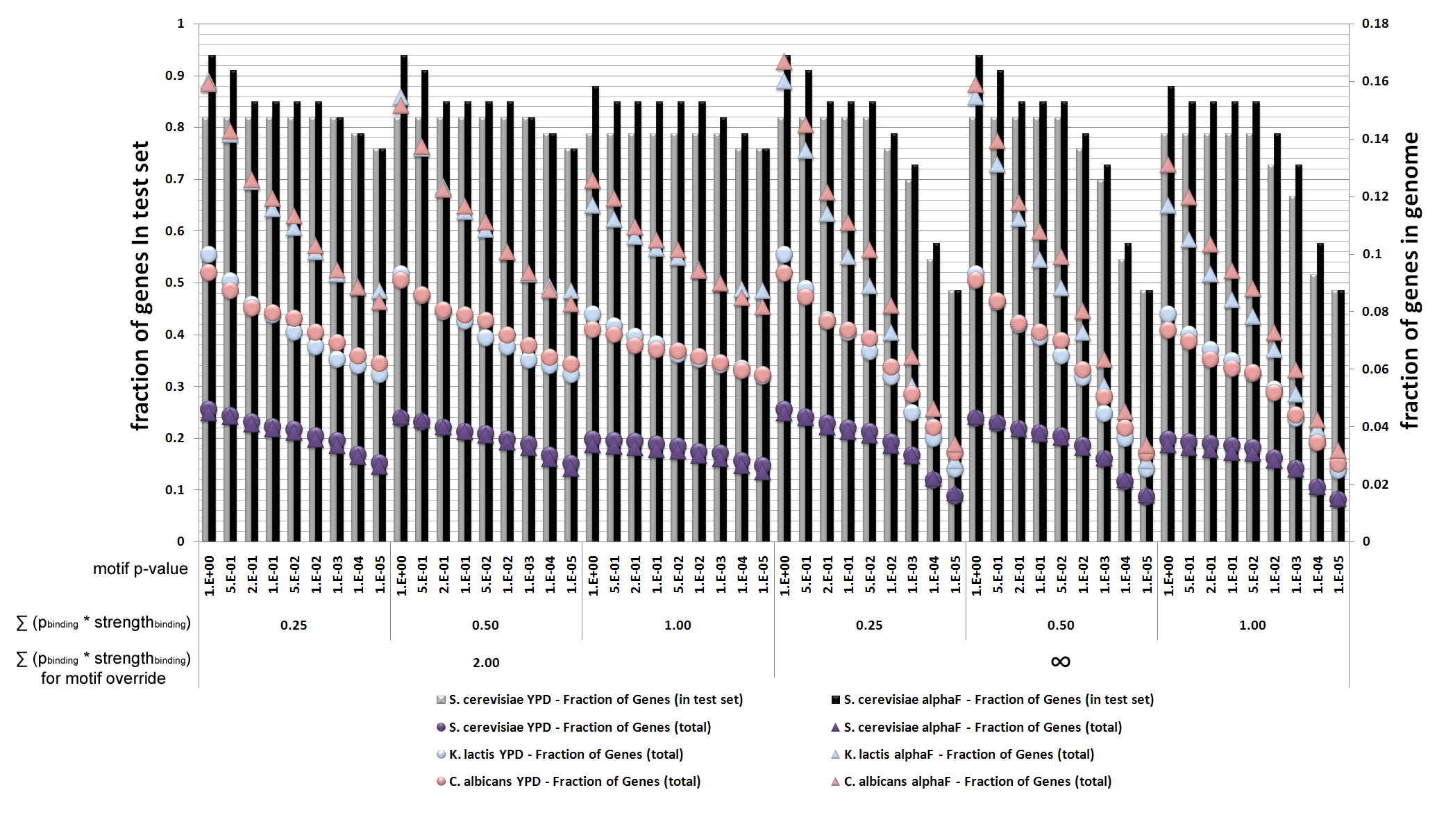

Supplement: Figure S8 — The cutoffs for the motif p-value and the JBD statistics (∑[p binding * strength binding] and ∑[p binding * strength binding] for motif override) were varied (x-axis), and the resulting number of bound genes called was recorded, both as a fraction of all test set genes in S. cerevisiae (left y-axis; silver and black bars) and as a fraction of all genes in each of the three genomes (right y-axis; pink, purple, and blue lines). Here the cutoff for p binding is 0.2. (1.2 MB DOC) [file pbio.0060038.sg008.doc]
