# Supplementary material for: The Evolution of Combinatorial Gene Regulation in Fungi
Source: PLoS Biol. 2008 Feb 26;6(2):e38. doi: 10.1371/journal.pbio.0060038 (PMC2253631; doi:10.1371/journal.pbio.0060038)
Supplement: Table S2 — (91 KB DOC) [file pbio.0060038.st002.doc]

| **Species** | **Source** |
| --- | --- |
| S. cerevisiae | [97] |
| S. paradoxus | [98] |
| S. mikatae | [98] |
| S. bayanus | [98] |
| S. castellii | [99] |
| C. glabrata | [97] |
| K. waltii | [98] |
| S. kluyveri | [99] |
| K. lactis | [97] |
| E. gossypii | [97] |
| C. dubliniensis | [100] |
| C. albicans | [101,102] |
| C. tropicalis | [103] |
| C. parapsilosis | [100] |
| L. elongisporus | [103] |
| C. guilliermondii | [103] |
| D. hansenii | [97] |
| C. lusitaniae | [103] |
| Y. lipolytica | [97] |
| A. terreus | [103] |
| A. nidulans | [103] |
| H. capsulatum | [103] |
| U. reesii | [103] |
| C. immitis | [103] |
| F. graminearum | [103] |
| T. reesei | [104] |
| M. grisea | [103] |
| C. globosum | [103] |
| N. crassa | [103] |
| S. sclerotiorum | [103] |
| S. japonicus | [103] |
| S. pombe | [97] |
