# Supplementary material for: The Evolution of Combinatorial Gene Regulation in Fungi
Source: PLoS Biol. 2008 Feb 26;6(2):e38. doi: 10.1371/journal.pbio.0060038 (PMC2253631; doi:10.1371/journal.pbio.0060038)
Supplement: Table S3 — (145 KB DOC) [file pbio.0060038.st003.doc]

| **Gene** | **ORF Id** | **Source DB** | **Literature Source** |
| --- | --- | --- | --- |
| ACE2 | YLR131C | -- | [105] |
| AGA1 | YNR044W | YPD | [106] |
| AGA2 | YGL032C |  | [107] |
| ARG1 | YOL058W | -- | [108] |
| ARG5,6 | YER069W | YPD | [109] |
| ASE1 | YOR058C | -- | [110] |
| ASG7 | YJL170C | YPD | [107] |
| BAR1 | YIL015W | YPD | [107,111] |
| BUD4 | YJR092W | -- | [112] |
| CAR1 | YPL111W | YPD | [109] |
| CAR2 | YLR438W | YPD | [109] |
| CCP1 | YKR066C | SCPD | [113] |
| CDC20 | YGL116W | -- | [114] |
| CDC46 | YLR274W | YPD | [115] |
| CDC47 | YBR202W | YPD | [115] |
| CDC5 | YMR001C | YPD | [116] |
| CDC6 | YJL194W | YPD | [115] |
| CLB1 | YGR108W | YPD | [116] |
| CLB2 | YPR119W | YPD | [116] |
| CLN3 | YAL040C | TRANSFAC | [117] |
| FAR1 | YJL157C | SCPD | [118] |
| HSP150 | YJL159W | SCPD | [113] |
| MCM3 | YEL032W | YPD | [114] |
| MFA1 | YDR461W | SCPD | [119] |
| MFA2 | YNL145W | -- | [119] |
| PCK1 | YKR097W | SCPD | [113] |
| PIS1 | YPR113W | SCPD | [113] |
| PMA1 | YGL008C | YPD | [113] |
| SPS4 | YOR313C | -- | [120] |
| STE2 | YFL026W | YPD | [107,111] |
| STE6 | YKL209C | YPD | [121] |
| SWI4 | YER111C | YPD | [115] |
| SWI5 | YDR146C | YPD | [116] |

105. Pic A, Lim FL, Ross SJ, Veal EA, Johnson AL, et al. (2000) The forkhead protein Fkh2 is a component of the yeast cell cycle transcription factor SFF. Embo J 19: 3750-3761.

106. Mead J, Bruning AR, Gill MK, Steiner AM, Acton TB, et al. (2002) Interactions of the Mcm1 MADS box protein with cofactors that regulate mating in yeast. Mol Cell Biol 22: 4607-4621.

107. Zhong H, McCord R, Vershon AK (1999) Identification of target sites of the alpha2-Mcm1 repressor complex in the yeast genome. Genome Res 9: 1040-1047.

108. Yoon S, Govind CK, Qiu H, Kim SJ, Dong J, et al. (2004) Recruitment of the ArgR/Mcm1p repressor is stimulated by the activator Gcn4p: a self-checking activation mechanism. Proc Natl Acad Sci U S A 101: 11713-11718.

109. Messenguy F, Dubois E, Boonchird C (1991) Determination of the DNA-binding sequences of ARGR proteins to arginine anabolic and catabolic promoters. Mol Cell Biol 11: 2852-2863.

110. Juang YL, Huang J, Peters JM, McLaughlin ME, Tai CY, et al. (1997) APC-mediated proteolysis of Ase1 and the morphogenesis of the mitotic spindle. Science 275: 1311-1314.

111. Galgoczy DJ, Cassidy-Stone A, Llinas M, O'Rourke SM, Herskowitz I, et al. (2004) Genomic dissection of the cell-type-specification circuit in Saccharomyces cerevisiae. Proc Natl Acad Sci U S A 101: 18069-18074.

112. Sanders SL, Herskowitz I (1996) The BUD4 protein of yeast, required for axial budding, is localized to the mother/BUD neck in a cell cycle-dependent manner. J Cell Biol 134: 413-427.

113. Kuo MH, Grayhack E (1994) A library of yeast genomic MCM1 binding sites contains genes involved in cell cycle control, cell wall and membrane structure, and metabolism. Mol Cell Biol 14: 348-359.

114. Pramila T, Miles S, GuhaThakurta D, Jemiolo D, Breeden LL (2002) Conserved homeodomain proteins interact with MADS box protein Mcm1 to restrict ECB-dependent transcription to the M/G1 phase of the cell cycle. Genes Dev 16: 3034-3045.

115. Fitch MJ, Donato JJ, Tye BK (2003) Mcm7, a subunit of the presumptive MCM helicase, modulates its own expression in conjunction with Mcm1. J Biol Chem 278: 25408-25416.

116. Althoefer H, Schleiffer A, Wassmann K, Nordheim A, Ammerer G (1995) Mcm1 is required to coordinate G2-specific transcription in Saccharomyces cerevisiae. Mol Cell Biol 15: 5917-5928.

117. McInerny CJ, Partridge JF, Mikesell GE, Creemer DP, Breeden LL (1997) A novel Mcm1-dependent element in the SWI4, CLN3, CDC6, and CDC47 promoters activates M/G1-specific transcription. Genes Dev 11: 1277-1288.

118. Oehlen LJ, McKinney JD, Cross FR (1996) Ste12 and Mcm1 regulate cell cycle-dependent transcription of FAR1. Mol Cell Biol 16: 2830-2837.

119. Zhong H, Vershon AK (1997) The yeast homeodomain protein MATalpha2 shows extended DNA binding specificity in complex with Mcm1. J Biol Chem 272: 8402-8409.

120. Hollenhorst PC, Pietz G, Fox CA (2001) Mechanisms controlling differential promoter-occupancy by the yeast forkhead proteins Fkh1p and Fkh2p: implications for regulating the cell cycle and differentiation. Genes Dev 15: 2445-2456.

121. Gavin IM, Kladde MP, Simpson RT (2000) Tup1p represses Mcm1p transcriptional activation and chromatin remodeling of an a-cell-specific gene. Embo J 19: 5875-5883.
