# Supplementary material for: The Evolution of Combinatorial Gene Regulation in Fungi
Source: PLoS Biol. 2008 Feb 26;6(2):e38. doi: 10.1371/journal.pbio.0060038 (PMC2253631; doi:10.1371/journal.pbio.0060038)
Supplement: Text S1 — (242 KB DOC) [file pbio.0060038.sd002.doc]

Supporting Method – Tiling array design

We designed three custom ChIP-Chip arrays by tiling 181,900 probes of length 60 bp across the:

1. 12.1 Mb of sequence included in the GenBank release of the *S. cerevisiae* genome dated 5/12/2006 (downloaded 5/2006)
2. 10.7 Mb of sequence included in the GenBank release of the *K. lactis* genome dated 7/30/2004 (downloaded 5/2006)
3. 14.3 Mb of sequence included in what, at the time of design, was the most recent build of the *C. albicans* genome (Assembly 20 from Andre Nantel 4/2006).

Rather than choose probes spaced uniformly, an effort was made to optimize four characteristics of the oligo set (uniqueness, GC content, self-annealing and sequence complexity), while still maintaining probe spacing that is close to uniform. We used a previously developed algorithm, ArrayOligoSelector[68], to score all possible probes for each of these four characteristics. These scores, as well as a penalty for a too-long or too-short distance to the neighboring probe, were integrated into a single score via a weighting scheme. Due to the lack of studies which systematically explore the importance of uniform spacing and each of the four probe characteristics on ChIP-chip quality, we chose weights based largely on intelligent guessing. Stronger weights were given to probe spacing, GC content and uniqueness than to self-annealing and sequence complexity. A Monte Carlo optimization was employed to search for the highest scoring probe set.

The highest scoring probe sets found for *S. cerevisiae, K. lactis* and *C. albicans* had median probe spacing (measured from oligo start to oligo start) of 66 bp, 59 bp, and 79 bp respectively (Figure S1). The uniqueness of the probe set chosen for each genome (pink curve) was significantly higher than that for all possible probes in the genome (red curve). A higher uniqueness score indicates a more unique probe that is less likely to be affected by cross-hybridization. For *C. albicans*, whereas only 27% of all possible probes are completely unique (uniqueness score of 0.0), 40% of the probes in our probe set are completely unique. Note that the tail of this distribution (uniqueness score < 35) has been truncated in the graph. Finally, the GC content of the chosen oligos (pink curve) was much improved over the genome as a whole (red curve). While the average was kept similar to that of the whole genome, the variance was considerably reduced.

Supporting Method – Mcm1 ChIP-Chip experiments

*Strains and Media***.** The following strains were used in our ChIP-Chips of the three species:

| **Species** | **Genotype** | **Strain Id** |
| --- | --- | --- |
| *S. cerevisiae* | S288c *MATa* prototroph | yDG765 |
| *K. lactis* | *MATa lysA1 trp1 leu2 metA1 uraA1* | SAY45[69] |
| *C. albicans* | SC5314*: ura3::imm434/ ura3::imm434 iro1::imm434/ iro1::imm434 mtlα1Δ::hisG-URA3-hisG mtlα2Δ::hisG* | RRY8 (white isolate)  yDG914 (opaque isolate of RRY8) |
| C. albicans | ade2::hisG/ade2::hisG ura3::imm434/ura3::imm434 ENO1/eno1::ENO1-tetR-ScHAP4AD-3xHA-ADE2 pTR(97t)-CaMCM1-Myc-URA3/camcm1::FRT | MRcan42[70] |

For growth in YEPD, cells were grown at 30°C overnight to an OD_600_ of 0.4. For growth in α-factor, cells were treated as needed for efficient pheromone response in the three species as follows. The *S. cerevisiae* strain *(yDG765)* was grown in YEPD at 30°C overnight to an OD600 of 0.4, synthetic α-factor (Sigma-Aldrich) was added to a final concentration of 50nM (in water), and treated cells were incubated for 30min, shaking. For *K. lactis (SAY45)*, cells were grown overnight at 30°C in SCD medium supplemented with 500μg/ml leucine to an OD600 of 0.9. Cells were then pelleted, washed twice in sterile water, resuspended in 200ml SCD medium lacking phosphate and supplemented with 500μg/ml leucine at an OD600 of 0.25, and incubated 6hr at 30°C. 165μl 13-mer α-factor (WSWITLRPGQPIF; Genemed Synthesis, So. San Francisco, Ca; 10mg/ml in 10% DMSO) was added and cells were grown for 4hr at 30°C. For *C. albicans (yDG914)*, five opaque colonies were taken from a synthetic complete with 2% dextrose and 100μg/ml uridine (SCD + Urd) plate and grown in YEPD for
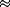
16h at 25°C to an OD_600_ of 0.4. Cells were pelleted, washed twice in sterile water, resuspended in 200ml SpiderM medium[71] at an OD600 of 0.4. Cells were then treated with 13mer α-factor[72] at a final concentration of 10μg/ml (from 10mg/ml stock in 10% DMSO), and incubated 4hr at 25°C.

*Chromatin Immunoprecipitation.* For chromatin immunoprecipitation, formaldehyde (37%) was added to a 1% final concentration. Treated cultures were shaken to mix and incubated for 15min at room temperature. 2.5M glycine was added to a final concentration of 125mM, and treated cultures were mixed and incubated 5min at room temperature. Cells were pelleted at 3,000 x g for 5min at 4°C and washed twice with 100ml 4°C TBS (20mM TrisHCl, pH7.6/150mM NaCl). Spheroplasting and ChIP were carried out as previously described[73], with modifications. Cell pellets were resuspended in 20 ml Buffer Z + β-ME (1 M sorbitol, 50 mM Tris-Cl [pH 7.4], 10 mM β-ME) and cells were vortexed. *S. cerevisiae*, *K. lactis*, or *C. albicans* cell suspensions were lysed using 500μl of zymolyase (5mg/ml in Buffer Z) or 2μl or 20μl of lyticase (Sigma, MO, United States) solution (2 mg/ml in Buffer Z), respectively. Cell suspensions were incubated for 30min (*S. cerevisiae* and *K. lactis*) or 15min (*C. albicans*) at 30°C. Spheroplasted cells were then spun at 3,000 × g, 10 min, at 4°C and resuspended in 500μl 4°C lysis buffer (50 mM HEPES-KOH, pH 7.5,140 mM NaCl, 1 mM EDTA, 1% Triton X-100, 0.1% sodium deoxycholate) with protease inhibitors. All subsequent ChIP and wash steps were done at RT with 4°C buffers. DNA was sheared by sonication 10 times for 10 seconds at power setting 2 on a Branson 450 microtip sonicator (Danbury, CT), incubating on ice for 2 minutes between sonication pulses. Extracts were clarified by centrifugation. 50μl of extract were set aside as ChIP input material. For chromatin IPs, 450 μl lysis buffer was added to 50μl extract, and the appropriate Mcm1 antibody was added in the following quantities: 12μl antibody serum raised against a *S. cerevisiae* Mcm1 peptide[74]; 15μl affinity purified antibody raised against a C-terminal peptide from *K. lactis* Mcm1 (Bethyl Laboratories, Montgomery, TX); or 5μl affinity purified antibody raised against N- or C- terminal peptides from *C. albicans* Mcm1 (Bethyl Laboratories, Montgomery, TX). Extract plus antibody was incubated 2hr at 4°C, with agitation. 50μl of a 50% suspension of protein A-Sepharose Fast-Flow beads (Sigma, St. Louis, MO) in lysis buffer was added and incubated 1.5hr at 4°C, with agitation. The beads were pelleted 1min at 3,000 × g. After removal of the supernatant, the beads were washed with a series of buffers for five minutes for each wash: twice in lysis buffer, twice in high salt lysis buffer (50 mM HEPES-KOH, pH 7.5, 500 mM NaCl, 1 mM EDTA, 1% Triton X-100, 0.1% sodium deoxycholate), twice in wash buffer (10 mM Tris-HCl [pH 8.0], 250 mM LiCl, 0.5% NP-40, 0.5% sodium deoxycholate, 1mM EDTA), and once in TE (10 mM Tris, 1 mM EDTA [pH 8.0]). After the last wash, 100μl of elution buffer (50 mM Tris/HCl [pH 8.0], 10 mM EDTA, 1% SDS)) was added to each sample, and the beads were incubated at 65 °C for 15 min. The beads were spun for 1 min at 10,000 × g, and the supernatant was removed and retained. A second elution was carried out with 150μl elution buffer 2 (TE, 0.67% SDS) and eluates from the two elution steps were combined. For the ChIP input material set aside earlier, 200μl TE, 1%SDS was added. ChIP and input samples were incubated overnight at 65°C to reverse crosslinks. 250μl proteinase K solution (TE, 20μg/ml glycogen, 400μg/ml Proteinase K) was added to each sample, and samples were incubated at 37°C for 2h. Samples were extracted once with 450μl Tris buffer-saturated phenol/chloroform/isoamyl alcohol solution (25:24:1). 55μl 4M LiCl and 1ml 100% ethanol (4°C) was added and the DNA was precipitated on ice for 1hr. The DNA was pelleted by centrifugation at 14,000 × g for 15 min at 4 °C, washed once with cold 75% ethanol, and allowed to air dry. Samples were resuspended in 25μl TE containing 100μg/ml RNaseA and incubated 1hr at 37°C.

For the verification of Mcm1 binding at the non-canonical motif in the myc-tagged Mcm1 strain (MRcan42) the protocol above was used with the following modifications. Cells were lysed by bead mixing rather than by spheroplasting. After washing with TBS, cell pellets were resuspended in 700μl ice-cold lysis buffer with protease inhibitors and 500μl of 0.5mm glass beads was added. This mix was placed in an Eppendorf mixer (part #5432) for 2h at 4°C. Chromatin was sheared by sonication, as before, but in this case with a Bioruptor (Wolf Laboratories, Manchester, UK) for 15min (30s on, 60s off) on the medium setting. Here the IP was carried out with anti-myc antibody 9E10 (Invitrogen, Carlsbad, CA) and protein G-Sepharose Fast-Flow beads (Sigma, St. Louis, MO).

*DNA amplification and labeling.* ChIP-enriched DNA was amplified and fluorescence labeled as described[75]. Labeled DNA for each channel was combined and hybridized to arrays in Agilent hybridization chambers for 40 hours at 65°C, according to protocols supplied by Agilent (Agilent Technologies, Santa Clara, CA). Arrays were then washed and scanned, using an Axon Instruments Genepix 4000A scanner.

Supporting Method – Identification of binding events in ChIP-Chip data

We evaluated several approaches for calling Mcm1 binding sites from the ChIP-Chip data in three species. In the end we determined that the Joint Binding Deconvolution[76] (JBD) algorithm provides the best combination of consistency across species and accuracy on a test set of previously characterized *S. cerevisiae* binding sites. In what follows we compare two methods for defining binding sites based on ChIP-Chip data:

1. A software package from Agilent called Chip Analytics v 1.3 (CA). This software first applies the single array error model (SAEM; first introduced by Roberts et al.[77] for analysis of gene expression microarray data and subsequently used for ChIP-Chip analysis by Ren et al.[78]) to calculate an enrichment statistic (X_bar_), which is a normalized comparison of signal in the Immuno-Precipitate (IP) channel to signal in the Whole Cell Extract (WCE) channel for every probe on the array. The distribution of X_bar_ values is then fit with a Gaussian by taking the mean and standard deviation over the entire distribution. Next, a p-value is assigned to each probe based on the placement of that probe’s enrichment statistic on the fitted Gaussian distribution. “Segments” (regions likely to be bound by the IP-ed protein) are then called by a peak identification heuristic called the “Whitehead Per-Array Neighbourhood Model v1.0”, which looks for neighboring probes with p-values below a threshold. Here we use the program’s default parameters with the exception of the parameter specifying “maximum distance (in bp) for two probes to be considered as neighbors”, which we set to 500bp rather than 1000bp. In testing this algorithm we vary only a single parameter, the p-value threshold for inclusion of a probe in a segment.
2. A software package from the Fraenkel Group called Joint Binding Deconvolution (JBD). The package takes a somewhat more sophisticated approach to the problem and is described in detail elsewhere[76]. Briefly, JBD treats the observed enrichment ratios as a convolution of unobserved discrete binding events and an “influence” function derived from the distribution of DNA shear lengths. JBD attempts to deconvolute these, producing probabilities of binding at or below the resolution of the tiling array. Here again we use the program’s default settings varying two parameters p_binding_ and ∑(p_binding_ * strength_binding_) to define bound regions. Prior to processing with JBD we perform a global loess normalization for each experimental replicate using Goulphar[79] (exact options are foreground=0, do.bgcorr=1, do.saturating=1, saturating=55000). A requirement of the JBD algorithm is an estimate of the influence function for each experimental replicate. We estimate this influence function from the data as described later in “Estimating influence functions for JBD”.

We compared the two methods (CA and JBD) over a broad range of parameter choices on the two ChIP-Chip datasets from *S. cerevisiae* (“YPD” and “alphaF”). As shown in the receiver operating characteristic (ROC) plot below (Figure S2), both methods perform similarly on our test set of previously characterized Mcm1 bound genes from *S. cerevisiae* (see “A test set of Mcm1 regulated *S. cerevisiae* genes”). For example, there are some parameter settings for each algorithm that call ~94% of test set genes bound while also calling only ~4% of all genes bound in S*. cerevisiae*. In the absence of a test set of non-bound genes, we think this is the most appropriate way to evaluate performance of the algorithm.

These results suggest that the two algorithms perform equally well. As one might expect then, genes called as bound outside of the test set are also very similar between the algorithms. With this result we decided to move forward with an analysis on all three species using the much less computationally intensive CA algorithm.

Unfortunately, the success seen with the Chip Analytics algorithm in *S. cerevisiae* was not reproduced on the data from *K. lactis* and *C. albicans*. Due to the lack of published experiments, it is not feasible to put together test sets for *K. lactis* and *C. albicans*. Nevertheless, problems became apparent in our attempts to choose a single p-value threshold across species, as shown in the plot below (Figure S3). Here we compare the gene sets resulting from a variety of enrichment p-value parameters both within the *S. cerevisiae* test set (left axis; silver and black bars) and across the three genomes (right axis; pink, purple and blue lines). Our expectation was that lower values for this threshold would result in proportionally smaller sets of genes called as bound. However, a strange behavior is found especially for the *K. lactis* and *C. albicans* alphaF experiments, where lowering of the enrichment p-value threshold results in a disproportionate loss of Mcm1 targets compared to the loss in other experiments (e.g. compare the drop in “fraction of the genes in genome” bound by Mcm1 resulting from a drop in p-value threshold from 10^-2^ to 10^-3^ in the *C. albicans* YPD and alphaF experiments). This behavior suggests that the calculation of these p-values may be flawed.

Examination of the distributions of the enrichment statistic (X_bar_) calculated from the SAEM reveals the underlying problem (Figure S4). For *S. cerevisiae* the assumption made by the SAEM that most probes on the array are not enriched is valid and the corresponding SAEM Gaussian fit (Figure S4a blue curve) is reasonable. However, this assumption is clearly not valid for *K. lactis* and *C. albicans* where a Gaussian distribution for a subset of the probes is evident, but where a sizeable portion of the X_bar_ distribution is found in the tail (Figure S4b,c). As expected, the Gaussian fit provided by Chip Analytics (estimated from the mean and standard deviation of all X_bar_ values) for these data is highly suspect. That SAEM is problematic on datasets where >5% of probes show enrichment was previously recognized[80]. Because the shape of the X_bar_ distribution varies so much between experiments, we can not rely on this method to obtain p-values.

We attempted to remedy this problem by performing a least-squares fit of the data to a Gaussian in which the overall weight was not fixed at 1.0 (this method is referred to hereafter as “CA_FIX”). In other words, a fit to the following equation:

While this approach does give an improved fit to the non-enriched portion of the data (Figure S4a,b,c red lines), strange behavior is still evident when applied across experiments (Figure S5). Although on the surface it would appear that we have corrected the scaling problem seen in Figure S3, examining plots of the ChIP enrichment for the YPD and alphaF experiments across the *C. albicans* genome reveals a new problem. For these two experiments CA_FIX predicts roughly equal numbers of bound genes across all p-value thresholds chosen. However, in a quick visual scan across the genome it is apparent that there are roughly 50-100% more Mcm1 binding events in the *C. albicans* alphaF experiment than in the *C. albicans* YPD experiment (plots online at http://genome.ucsf.edu/mcm1_evolution/). Perhaps there is no simple remedy to the SAEM approach when a large fraction of probes on the array is enriched.

In principle, the fatter tail exhibited in the X_bar_ distributions from the *K. lactis* and *C. albicans* alphaF experiments can be attributed to either of two sources: (1) a larger number of Mcm1 binding events or (2) a longer/wider DNA shear length distribution. A visual scan of the enrichment data plotted along the chromosomes suggests that there is indeed a larger number of Mcm1 binding events in the *K. lactis* and *C. albicans* alphaF experiments than in the other experiments. Comparison of the estimated influence functions for each experiment (Figure S6; also see “Supplementary Methods – Estimation of influence functions for JBD”) and examination of the whole-cell extract DNA lengths on a gel (not shown), indicate that the shearing of DNA is probably not as complete in *K. lactis* and *C. albicans* alphaF experiments as it is in some of the other experiments. Therefore, in our experiments both effects are likely contributing to the substantial fraction of probes enriched in the IP.

Because our data are ill-suited for the SAEM analysis of CA, we turn to JBD, which performs equally well on the *S. cerevisiae* test set, but also has the advantage that it directly accounts for the variability of DNA shear distributions through its use of influence functions. As the plot below (Figure S7) shows, JBD gives us the scaling we expect across different parameter values, while also agreeing with our expectations from visual inspection of the data plotted across the genome (e.g. that there are roughly 50-100% more binding events in the *K. lactis* alphaF experiment than in the *K. lactis* YPD experiment).

Supporting Method – Integration of motif information and the final Mcm1-bound segment calls

Mcm1 binds a well defined *cis* regulatory motif in *S. cerevisiae*[81,82]. *De novo* motif finding with MEME[83] on sequences predicted by JBD to be bound with high confidence (p_binding_ > 0.9 and ∑[p_binding_*strength_binding_] > 2.0) gives Mcm1 motifs that are roughly the same in each species (see Figure 1). We decided to integrate this motif information into our Mcm1-bound segment calls in the following manner. The motif matrices found by MEME for the YPD ChIP-Chip experiments for each species were used to score overlapping 1 kb windows across the corresponding genome, taking the sum of all the odds ratios against the matrix in each window as the “motif score” for a window. The distribution of log_10_ motif scores was fit with a Gaussian, taking the mean and the variance of log_10_ motif scores across the genome. Motif p-values for each genomic window were calculated on this Gaussian distribution. For *C. albicans*, where we find Mcm1 associating with two different *cis* regulatory motifs (Figure 1), we modified the above method so that each 1kb window is scored with both the canonical and non-canonical Mcm1 motif matrices and the odds ratios of each across the window are summed.

In our final Mcm1-bound segment calls we integrated four parameters (one which defines the contribution of Mcm1 sequence motifs and three that define the contributions of ChIP-Chip enrichment):

1. motif p-value; this parameter, on the scale 0.0 to 1.0, determines the weight given to the presence of Mcm1 motifs within the segment and can be overridden by parameter (4).
2. p_binding_; this parameter, on the scale 0.0 to 1.0, determines the minimum probability of binding as output by JBD.
3. ∑(p_binding_*strength_binding_); this parameter, on the scale 0.0 to ∞, determines the minimum “sum of probability of binding times strength of binding” as output by JBD.
4. ∑(p_binding_*strength_binding_) override; if the ∑(p_binding_*strength_binding_) is larger than this parameter then the region is called as Mcm1-bound regardless of motif p-value.

Parameters (2) and (3) were suggested by the developers of JBD as a reasonable way to define bound regions[76]. The fourth parameter was added when we observed that occasionally strong Mcm1 enrichment is unaccompanied by an Mcm1 motif, possibly due to errors in genome sequencing in the bound region or possibly due to recruitment of Mcm1 to promoters by other transcription factors.

A large number of parameter choices were sampled and the resulting *S. cerevisiae* test set accuracies for a subset of these choices (where p_binding_≥0.2) are plotted in Figure S8 (left axis; silver and black bars). Additionally, the fraction of genes in each genome called as bound was reported for each experimental condition (right axis; pink, purple and blue lines). In the end we chose the following parameter cutoffs to define bound regions in each species:

motif p-value 0.1

p_binding_ 0.2

∑(p_binding_*strength_binding_) 0.5

∑(p_binding_*strength_binding_) override 2.0

Supporting Method – Estimating the rate of false positive Mcm1 binding site calls

Although the JBD algorithm performs more consistently on our Mcm1 datasets (as explained above), one deficiency of this program is its inability to estimate false positive rates. The authors of the JBD algorithm suggest two ways of estimating false positive rates (http://cgs.csail.mit.edu/jbd/signif.html). The first relies on a set of regions where it is known *a priori* that no binding events occur; this we do not have. The second method relies on scrambling the data for each probe with respect to the chromosomal coordinate, which we think is not a particularly accurate way of estimating false positives because it destroys much of the long range correlation structure of ChIP-Chip experiments that would tend to give rise to false positives in the first place.

Assuming that only those regions with both ChIP-Chip enrichment and Mcm1 binding site motifs represent the bona fide *cis*-acting sequences, we estimate that using the ChIP-Chip data alone yields false positive rates between 11 and 36%. This can be calculated from Figure S8 by comparing the “fraction of genes in the genome” bound when using our chosen parameters, where the motif p-value cutoff is 0.1, to those when the motif p-value cutoff is 1.0 (i.e., when motif matches are not considered). Because our motif p-values are derived by fitting the genome-wide motif score distribution to a Gaussian (explained in the previous section), applying our motif p-value cutoff of 0.1 to a set of randomly chosen genomic regions would reduce the number of regions by ~90%.

With the added requirement of an Mcm1 binding site motif under the ChIP-Chip peaks, we believe our false positive rates are likely much lower. For experiments in *S. cerevisiae*, our false positive rate *before* integrating the motif information can be estimated using our test set as follows:

$$PP_{after}=FP_{before}\times EFPR+TP_{before}\times ETPR=FP_{before}\times EFPR+(PP_{before}-FP_{before})\times ETPR$$

where PP is the number of predicted positives and FP and TP are the numbers of false and true positives, respectively. An upper estimate of the rate at which false positives pass the motif filter (EFPR) is 0.1. The rate at which true positives pass the motif filter (ETPR) is estimated by the fraction of our test set remaining after motif filtering (28/31 = 0.9). Solving for FP_before_, gives an estimate of ~7 false positives *before* filtering and thus < 1 (7 * 0.1) false positive *after* filtering.

Supporting Method – Estimation of influence functions for JBD

An influence function is used by JBD to specify “the expected relative probe intensity as a function of distance from a binding event”[76]. In their paper Qi et al. derive the influence function from the distribution of shear fragment lengths. Here we estimate influence functions for each experiment by averaging the relative enrichment as a function of distance for the 50 strongest, idealized peaks in each experiment (Figure S6). Specifically, we start by sorting IP/WCE ratios for all probes (normalized with Goulphar as described above). We then move in descending order through the list and for each candidate probe look in the genomic region ±3kb, defining the candidate peak probe as having ratio $\left(\frac{IP}{WCE}\right)_{0}$with position d=0. If (1) this flanking region does not contain a probe that was previously annotated as part of another peak and (2) this region contains one probe with at least half the enrichment of the peak probe:

i.e., $\frac{\left(\frac{IP}{WCE}\right)_{x}}{\left(\frac{IP}{WCE}\right)_{0}}\geq0.5$ for at least one probe x

and (3) the $\left(\frac{IP}{WCE}\right)_{x}$ratios continually decrease at more distant probes:

ie., $\frac{\left(\frac{IP}{WCE}\right)_{x}}{\left(\frac{IP}{WCE}\right)_{0}}$ can not be greater than 0.4 + $\frac{\left(\frac{IP}{WCE}\right)_{\min\text{ }at\text{ }d\leq x}}{\left(\frac{IP}{WCE}\right)_{0}}$

then the region is taken to be a peak and the relative enrichment levels, $\frac{\left(\frac{IP}{WCE}\right)_{x}}{\left(\frac{IP}{WCE}\right)_{0}}$, are recorded for each probe at distance x from the peak probe 0. The second criterion filters “peaks” consisting of a single probe (a.k.a. “blips”) and the third criterion is a heuristic that attempts to filter regions in which there is more than one binding event. The algorithm terminates when it has recorded relative enrichment levels for 50 idealized peaks. The relative enrichments at each distance x are averaged across the peaks. As the resulting influence function is somewhat rough and typically lacking data for many distances x, we smooth it by replacing each relative enrichment level at distance x with the average of all relative enrichment levels in the range x ± 50 bp. The influence function for each of our experiments is plotted in Figure S6.

Supporting Method – Inference of the 32 species fungal phylogeny

A robust phylogeny of the 32 yeast species was inferred using the methods similar to those previously developed[84,85].  To build the phylogeny, orthologous gene sets (see “Mapping orthologous gene sets”) containing one and only one representative from each of the 32 yeasts were chosen at a stringent branch length cutoff (0.5; see “Mapping orthologous gene sets” below).  Of the resulting 122 orthologous gene sets, 22 are affected by the phenomenon of differential gene loss following the WGD and were filtered[86].  This yielded 100 orthologous gene sets that, showing no evidence for deletion or duplication events, were more likely than other orthologous gene sets to preserve the underlying speciation signal.  The 32 sequences within each set were then multiply aligned with ClustalW[87]. The resulting 100 alignments were concatenated and columns containing gaps were dropped, producing a single alignment with 19,989 columns.  Finally, a maximum likelihood species tree was estimated employing the TREE-PUZZLE algorithm with default parameters (VT substitution model)[88].  Demonstrating the robustness of this inference, a tree with identical topology and similar branch lengths was generated when the neighbor-joining method of ClustalW[87] was applied to the same dataset (not shown). The maximum likelihood algorithm PHYML[89] (using the WAG substitution model as recommended by ProtTest[90]) also yields a similar tree that differs only in that *E. gossypii* branches with *K. lactis* rather than just prior to the whole clade that spans *S. cerevisiae* to *K. lactis*. If this alternate tree is correct it only serves to strengthen our argument for four independent gains of Mcm1 regulation at ribosomal genes. The alternate topology does not affect any of the other arguments we present in the paper. The alternate placement of *C.glabrata* (swapped with *S. castellii*) recently proposed by Scannell et al.[91] also serves to strengthen our argument for several independent gains of ribosomal gene regulation by Mcm1.

Supporting Method – Mapping orthologous gene sets (OGSs)

Here again we use a method similar to that which we used previously[84].  We ran PSI-BLAST for each *S. cerevisiae* ORF “query” sequence against a single database containing all ORF sequences from each of the 32 fungal species, employing an E-value cutoff of 10^-5^ and the Smith-Waterman alignment option[92].  The sequences returned by PSI-BLAST were then multiply aligned with MUSCLE (setting the maxhours parameter to 0.5 and maxiters to 2 if the sequence database was greater than 50,000 residues in length)[93] and a neighbor joining tree (NJ) was inferred, using ClustalW[87].  Finally, the resulting NJ tree was traversed to extract an orthologous gene set (OGS) in the following manner: Start at the leaf node for the query sequence and ascend the tree, incrementing a level counter for each node ascended.  At each internal node descend.  If a leaf node is reached, the gene is from a species not yet seen at a lower level, and the branch length traversed is less than a cutoff (1.0), then add that gene to the OGS. This procedure was repeated for each *S. cerevisiae* sequence, resulting in a 32 species many-to-many ortholog map.  For the purposes of generating Figure 2a,b and the section entitled “Genes bound in any one species are only moderately...” we attempted to reduce the species bias of this approach.  For these analyses a second OGS map was used in which additional OGSs were generated using each of the ORF sequences from *K. lactis* and *C. albicans* not already present in an OGS as a query sequence. Because our goal in the results section entitled, “Mcm1 binding at a non-canonical motif upstream…”, was to examine the presence of the non-canonical Mcm1 motif found in *C. albicans* at the orthologous promoters of other species, an ortholog map was built using the same method as above, but with the set of all *C. albicans* (rather than *S. cerevisiae*) ORF sequences serving as the query database.

As previously[84] we found that because the number of **a**sgs (7 in *S. cerevisiae* and 6 in *C. albicans*) and αsgs (5 in *S. cerevisiae*) is small, a more careful ortholog mapping of these genes benefits downstream analyses of promoter sequences.  For example, *MFA1* and *MFA2*, two **a**sgs from *S. cerevisiae*, are less than 40 amino acids long and were therefore not annotated as ORFs in several of the fungal sequencing projects.  Using TBLASTN we identified putative *MFA1/MFA2* orthologs and added them to our ortholog map.

Supporting Method – Robustness of results to parameter choices

In the Results section entitled “Genes bound in any one species are only moderately likely…” we claim that the results of our pairwise comparison of Mcm1 target gene sets between species are robust to the exact parameters chosen to define Mcm1 binding sites. In Figure S9 we support that claim with the results of the pairwise comparison of Figure 2a-b for a variety of parameter choices.

Supporting Method – Inference of Mcm1 binding site gain and loss rates

In order to assess the prevalence of gain and loss of Mcm1 binding sites across the three species phylogeny we constructed a 4 branch model with 9 parameters: 4 gain rates (g_1-4_) and 4 loss rates (l_1-4_) corresponding to each of the 4 branches of the rooted tree and a single parameter, p_1_, representing the probability of an Mcm1 binding site at the root of the tree (Figures 2c and S10). We take as our dataset the Mcm1 binding occurrence patterns at each of the 2766 genes that can be mapped between *S. cerevisiae*, *K. lactis*, and *C. albicans* in a 1:1:1 fashion via our ortholog mapping. There are eight such patterns, e.g. the pattern “101” for hypothetical gene X indicates an Mcm1 binding site is present upstream of gene X in *S. cerevisiae* and *C. albicans*, but not in *K. lactis*. The total counts for each occurrence pattern can be found in Figure 2c.

We use maximum likelihood approach to estimate the parameters. Since there are nine independent parameters and only seven independent occurrence patterns (eight patterns with the normalization that the sum equals the total number of genes), there is degeneracy in the solution. In fact, there is a two dimensional space of solutions with equal maximum likelihood, i.e., they all perfectly fit the observed patterns. It can be shown that the four branch rooted tree model is mathematically equivalent (in terms of the statistics of the occurrence patterns) to a three branch star model, where the center is the *S. cerevisiae*–*K. lactis* ancestor (node B in Figure S10), with branches 2 and 3 leading to *S. cerevisiae* and *K. lactis* and “branch” * leading to *C. albicans*. For the 3 branch model, there is a unique maximum likelihood solution, thus the parameters for branches 2 and 3 are fixed. The parameters for the * branch and the probability of an Mcm1 binding site at B are also fixed, and are related to the variable parameters for branch 1 and 4 and the ancestor in the 4 branch model, allowing us to explicitly find all possible solutions:

$$\begin{matrix} p_{B}=p(B=1)=p_{1}\left(1-l_{1}\right)+\left(1-p_{1}\right)g_{1} \\ g_{*}=\frac{p(B=0,C=1)}{p(B=0)}=\frac{p_{1}l_{1}\left(1-l_{4}\right)+\left(1-p_{1}\right)\left(1-g_{1}\right)g_{4}}{p_{1}l_{1}+\left(1-p_{1}\right)\left(1-g_{1}\right)} \\ l_{*}=\frac{p(B=1,C=0)}{p(B=1)}=\frac{p_{1}\left(1-l_{1}\right)l_{4}+\left(1-p_{1}\right)g_{1}\left(1-g_{4}\right)}{p_{1}\left(1-l_{1}\right)+\left(1-p_{1}\right)g_{1}} \end{matrix}$$

Solving the above equations for $l_{4}$ and $g_{4}$and substituting$p_{1}=\frac{p_{B}-g_{1}}{1-l_{1}-g_{1}}$ and $g_{1}=\frac{p_{B}-p_{1}\left(1-l_{1}\right)}{1-p_{1}}$ gives:

$$\begin{matrix} l_{4}=\frac{-l_{*}p_{B}+g_{1}\text{ }\left(1+g_{*}\left(p_{B}-1\right)+\left(l_{*}-1\right)p_{B}\right)}{g_{1}-p_{B}} \\ g_{4}=\frac{-l_{1}\left(l_{*}-1\right)\text{ }p_{B}+g_{*}\left(l_{1}+p_{B}-l_{1}\text{ }p_{B}-1\right)}{-1+l_{1}+p_{B}} \end{matrix}$$

Substituting our previously calculated 3-branch values for$g_{*}$, $l_{*}$ and $p_{B}$ gives the following equations in which the degeneracy of the four branch model is now clearly evident:

$$\begin{matrix} l_{4}=\frac{-0.03772+\text{0.90781 }g_{1}}{g_{1}-0.0563} \\ g_{4}=\frac{\text{0.05503 }l_{1}-0.0736}{l_{1}-0.9437} \end{matrix}$$

To estimate these four rates ($l_{1}$, $g_{1}$, $l_{4}$and$g_{4}$) and the probability of an Mcm1 binding site at the root of the tree ($p_{1}$), we chose the solution with minimal distance to all other equivalent ML solutions in this two dimensional space. We believe this is the most reasonable method for averaging with the constraint that the parameters chosen actually represent one of the ML solutions to the problem:

Supporting Method – Mcm1 DNA motifs are not present at genes that are not bound

Our argument that Mcm1 binding site turnover rates are high hinges on the completeness and reliability of our ChIP-Chip data. Here we attempt to demonstrate that genes which are not bound by Mcm1 in species A, but have an ortholog bound in another species B, also do not have evidence for Mcm1 *cis* regulatory elements in their promoters in species A. We find that 34% of genes bound by Mcm1 only in *S. cerevisiae* have Mcm1 motifs with log_10_-odds > 2.8 in *K. lactis* and *C. albicans*, a frequency roughly equivalent to the background rates of occurrence of 40% and 30% respectively (binomial p > 0.8 and p > 0.3 respectively). For *S. cerevisiae*, we find Mcm1 motifs at more than 67% of these promoters (employing the same log_10_-odds cutoff) as compared to a background rate of roughly 41% (binomial p < 10^-5^). Similar results are obtained when genes bound by Mcm1 only in *K. lactis* and only in *C. albicans* are examined in the other species.

Supporting Method – Mapping Mcm1-cofactor regulons across species

*Yox1, Fkh2,* ***a****-specific genes (****a****sgs).*Yox1 and Fkh2 regulons were identified in *S. cerevisiae* using Mcm1 ChIP-chip, cofactor ChIP-chip and cell-cycle gene expression data. Specifically, genes were taken to be part of the regulon if they were bound by Mcm1 in our ChIP-Chip experiments, bound by the cofactor in Harbison et al.’s ChIP-Chip experiments[94] and cell-cycle oscillating in Spellman et al.’s gene expression experiments[75]. For **a**sgs, the union of previously defined regulons in *S. cerevisiae* and *C. albicans* was used[84,95,96]. The **a**sg regulons of *S. cerevisiae* and *C. albicans* are based on ChIP-Chip, mating-type gene expression data and promoter sequence scoring against MATα2-Mcm1 and MATa2-Mcm1 motif matrices.

Regulons defined in *S. cerevisiae* were mapped to *K. lactis* and *C. albicans* via our standard ortholog map (see “Mapping orthologous gene sets”). In each species, bound segments (±250bp) flanking genes in the mapped regulon and promoters (600bp upstream of the translational start) of orthologous genes in closely related species were scored for the presence of a single Mcm1 binding site sequence with log-odds score greater than 2.0. Here we use the same position weight matrices previously used to define the bound segments (Figure 1).

- For *S. cerevisiae*, orthologous promoters from *S. mikatae, S. bayanus, S. castellii and C. glabrata* were used.
- For *K. lactis*, orthologous promoters from *K. waltii, S. kluyveri and E gossypii* were used.
- For *C. albicans*, orthologous promoters from *C. dubliniensis, C. tropicalis, C. parapsilosis* and *L elongisporus* were used.

The resulting putatively Mcm1 bound subsequences and flanking sequence (to a final length of 40, centered on the Mcm1 motif) were submitted to MEME with a min length parameter of 25, a max length parameter of 40 and a target frequency of 0.5. The choice of the target frequency parameter is somewhat arbitrary, but is based on the notion that not all submitted sequences are expected to be true operators within the regulon. The resulting multi-species position weight matrix (with 0.25 pseudocounts added for each nucleotide in each column) was then used to score all the bound segments in that species. A new, single-species weight matrix was then generated from only those sequences within the species which score below a p-value threshold (see below for calculation of p-value and choice of threshold; again, 0.25 pseudocounts were added for each nucleotide in each column of the weight matrix). The bound segments within the species were then rescored with the single-species weight matrix and final regulon membership was determined based on thresholding on the single-species weight matrix p-value.

For the purposes of standardizing cutoffs across species/regulons, sequence scores genome-wide for a given Mcm1-cofactor weight matrix were fit to a Gaussian so that the p-values could be calculated for each score. A p-value cutoff of 10^-7^ was employed in defining members of the regulon in both the first (using the multi-species weight matrix) and second (using the single-species weight matrix) passes. This parameter was chosen to maximize the number of known **a**sg regulon members in *S. cerevisiae* and *C. albicans*[84]; with a cutoff of 10^-7^ we achieve a 93% sensitivity, correctly identifying the *S. cerevisiae* recombinational enhancer and 12 of 13 known **a**sgs as members of the regulon (*C. albicans* Ram2 is missed because it lacks Mcm1 enrichment in our experiments), while also achieving near perfect specificity (only 6 bound regions flank ORFs that were not previously implicated as **a**sgs: orf19.171/orf19.172, orf19.2308, orf19.7380/orf19.7381 from *C. albicans*, and Cdc6/Elo1, Cdc47, Mcm3 from *S. cerevisiae*).

*MATα1.* The α-specific gene regulon was defined as the union of previously defined MATα1 regulons from *S. cerevisiae* and *C. albicans*. These regulons are based on ChIP-chip and mating-type gene expression data[95,96]. The procedure is the same as for the Yox1/Fkh2/**a**sgs regulons, but with two modifications because αsgs are not expected to be bound by Mcm1 in our ChIPs of **a** cells and because αsg motifs are often found in multiple copies upstream of target genes:

1. Instead of restricting the motif search to Mcm1 bound segments, we used the promoters of all αsg orthologs.
2. The MEME search is carried out on all subsequences scoring greater than 2.0, rather than just the max scoring subsequence greater than 2.0.

*Arg81.* A couple complications arose in the analysis of the Arg81 regulon. First, there was some question as to whether Mcm1 is really bound upstream of arginine metabolic genes with Arg80/81 *in vivo*. Our ChIPs indicate little to no enrichment of Mcm1 at genes typically cited as members of this regulon. This issue is discussed in greater length in the Results section. Second, it appears as if the strict positioning of the Mcm1/Arg80 *cis* regulatory site relative to Arg81 found in *S. cerevisiae* (see Figure 4b) is not found in *C. albicans*. For these reasons we took a somewhat different approach to mapping this regulon. We mapped genes encoding enzymes in the metabolic neighborhood of arginine (YPL111W, YOL058W, YJL088W, YER069W, YOL140W, YLR438W, YJL071W, YHR018C, YMR062C, YOR303W and YJR109C) to orthologs in *K. lactis* and *C. albicans*. We then performed a MEME search on the promoters (500bp upstream of the translational start) for these genes and the promoters of orthologous genes in closely related species:

- For *S. cerevisiae*, orthologous promoters from *S. bayanus, S. castellii and C. glabrata* were used.
- For *K. lactis*, orthologous promoters from *K. waltii, S. kluyveri and E gossypii* were used.
- For *C. albicans*, orthologous promoters from *C. dubliniensis,* and *C. tropicalis* were used.

In *S. cerevisiae*, the expected Mcm1/Arg80-Arg81 motif was found (Figure 4b), but we could not use this motif matrix to score Mcm1 bound segments because Mcm1 does not appear to bind at this regulon in our *S. cerevisiae* ChIP-Chips. In *K. lactis* a similar motif was found. As with motifs found at the other regulons, we fit the genome-wide distribution of log-odds scores for this motif matrix with a Gaussian and then calculated p-values for the maximum scoring motif match at each *K. lactis* Mcm1 bound segment. A p-value cutoff of 10^-6^ was employed to define regulon membership, yielding 9 segments and 17 genes (including Arg1/3/8, Car1/2 and Gap1) as members. Finally, in *C. albicans* an Mcm1-like motif and Arg81-like motif were found in separate MEME rounds (Figure 4b). This suggests that while Arg81 and Mcm1 regulate arginine metabolic genes together in *C. albicans*, they do so via a mechanism that allows for relaxed spacing between the two transcription factors. We fit each genome-wide distribution of log-odds scores for these two motif matrices with a Gaussian and then calculated p-values for the maximum scoring motif match at each *C. albicans* Mcm1 bound segment. We examined the 13 segments which matched both matrices with p-value < 10­^-5^ and found that in 8 cases the two motif matches were spaced 15 to 39bp from each other. Thus these 8 segments, which flank the genes Arg1/3/4, Car1 and Cpa1/2, define the Mcm1-Arg81 regulon in *C. albicans*.

*Yhp1.* We could not identify an *S. cerevisiae* regulon from existing data.

Supporting Method – A test set of Mcm1 regulated *S. cerevisiae* genes

A test set of previously characterized Mcm1 regulated genes was compiled from primary and secondary sources (Table S3). Secondary sources include:

YPD (Yeast Proteome Database; <https://www.proteome.com/proteome/Retriever/index.html>)

SCPD (S. cerevisiae Promoter Database; <http://rulai.cshl.edu/SCPD/>)

TRANSFAC (<http://www.biobase.de/cgi-bin/biobase/transfac/start.cgi>)

Supporting References

68. Bozdech Z, Zhu J, Joachimiak MP, Cohen FE, Pulliam B, et al. (2003) Expression profiling of the schizont and trophozoite stages of Plasmodium falciparum with a long-oligonucleotide microarray. Genome Biol 4: R9.

69. Carter SD, Iyer S, Xu J, McEachern MJ, Astrom SU (2007) The role of nonhomologous end-joining components in telomere metabolism in Kluyveromyces lactis. Genetics 175: 1035-1045.

70. Rottmann M, Dieter S, Brunner H, Rupp S (2003) A screen in Saccharomyces cerevisiae identified CaMCM1, an essential gene in Candida albicans crucial for morphogenesis. Mol Microbiol 47: 943-959.

71. Bennett RJ, Johnson AD (2006) The role of nutrient regulation and the Gpa2 protein in the mating pheromone response of C. albicans. Mol Microbiol 62: 100-119.

72. Bennett RJ, Uhl MA, Miller MG, Johnson AD (2003) Identification and characterization of a Candida albicans mating pheromone. Mol Cell Biol 23: 8189-8201.

73. Zordan RE, Miller MG, Galgoczy DJ, Tuch BB, Johnson AD (2007) Interlocking Transcriptional Feedback Loops Control White-Opaque Switching in Candida albicans. PLoS Biol 5: e256.

74. Jarvis EE, Clark KL, Sprague GF, Jr. (1989) The yeast transcription activator PRTF, a homolog of the mammalian serum response factor, is encoded by the MCM1 gene. Genes Dev 3: 936-945.

75. Spellman PT, Sherlock G, Zhang MQ, Iyer VR, Anders K, et al. (1998) Comprehensive identification of cell cycle-regulated genes of the yeast Saccharomyces cerevisiae by microarray hybridization. Mol Biol Cell 9: 3273-3297.

76. Qi Y, Rolfe A, MacIsaac KD, Gerber GK, Pokholok D, et al. (2006) High-resolution computational models of genome binding events. Nat Biotechnol 24: 963-970.

77. Roberts CJ, Nelson B, Marton MJ, Stoughton R, Meyer MR, et al. (2000) Signaling and circuitry of multiple MAPK pathways revealed by a matrix of global gene expression profiles. Science 287: 873-880.

78. Ren B, Robert F, Wyrick JJ, Aparicio O, Jennings EG, et al. (2000) Genome-wide location and function of DNA binding proteins. Science 290: 2306-2309.

79. Lemoine S, Combes F, Servant N, Le Crom S (2006) Goulphar: rapid access and expertise for standard two-color microarray normalization methods. BMC Bioinformatics 7: 467.

80. Buck MJ, Lieb JD (2004) ChIP-chip: considerations for the design, analysis, and application of genome-wide chromatin immunoprecipitation experiments. Genomics 83: 349-360.

81. Wynne J, Treisman R (1992) SRF and MCM1 have related but distinct DNA binding specificities. Nucleic Acids Res 20: 3297-3303.

82. Passmore S, Elble R, Tye BK (1989) A protein involved in minichromosome maintenance in yeast binds a transcriptional enhancer conserved in eukaryotes. Genes Dev 3: 921-935.

83. Bailey TL, Elkan C (1994) Fitting a mixture model by expectation maximization to discover motifs in biopolymers. Proc Int Conf Intell Syst Mol Biol 2: 28-36.

84. Tsong AE, Tuch BB, Li H, Johnson AD (2006) Evolution of alternative transcriptional circuits with identical logic. Nature 443: 415-420.

85. Rokas A, Williams BL, King N, Carroll SB (2003) Genome-scale approaches to resolving incongruence in molecular phylogenies. Nature 425: 798-804.

86. Byrne KP, Wolfe KH (2005) The Yeast Gene Order Browser: combining curated homology and syntenic context reveals gene fate in polyploid species. Genome Res 15: 1456-1461.

87. Higgins DG, Sharp PM (1988) CLUSTAL: a package for performing multiple sequence alignment on a microcomputer. Gene 73: 237-244.

88. Schmidt HA, Strimmer K, Vingron M, von Haeseler A (2002) TREE-PUZZLE: maximum likelihood phylogenetic analysis using quartets and parallel computing. Bioinformatics 18: 502-504.

89. Guindon S, Gascuel O (2003) A simple, fast, and accurate algorithm to estimate large phylogenies by maximum likelihood. Syst Biol 52: 696-704.

90. Abascal F, Zardoya R, Posada D (2005) ProtTest: selection of best-fit models of protein evolution. Bioinformatics 21: 2104-2105.

91. Scannell DR, Byrne KP, Gordon JL, Wong S, Wolfe KH (2006) Multiple rounds of speciation associated with reciprocal gene loss in polyploid yeasts. Nature 440: 341-345.

92. Altschul SF, Madden TL, Schaffer AA, Zhang J, Zhang Z, et al. (1997) Gapped BLAST and PSI-BLAST: a new generation of protein database search programs. Nucleic Acids Res 25: 3389-3402.

93. Edgar RC (2004) MUSCLE: multiple sequence alignment with high accuracy and high throughput. Nucleic Acids Res 32: 1792-1797.

94. Harbison CT, Gordon DB, Lee TI, Rinaldi NJ, Macisaac KD, et al. (2004) Transcriptional regulatory code of a eukaryotic genome. Nature 431: 99-104.

95. Galgoczy DJ, Cassidy-Stone A, Llinas M, O'Rourke SM, Herskowitz I, et al. (2004) Genomic dissection of the cell-type-specification circuit in Saccharomyces cerevisiae. Proc Natl Acad Sci U S A 101: 18069-18074.

96. Tsong AE, Miller MG, Raisner RM, Johnson AD (2003) Evolution of a combinatorial transcriptional circuit: a case study in yeasts. Cell 115: 389-399.
