# Supplementary material for: The Evolution of Combinatorial Gene Regulation in Fungi
Source: PLoS Biol. 2008 Feb 26;6(2):e38. doi: 10.1371/journal.pbio.0060038 (PMC2253631; doi:10.1371/journal.pbio.0060038)
Supplement: Supplementary file 7 [file pbio.0060038.sg002.doc]

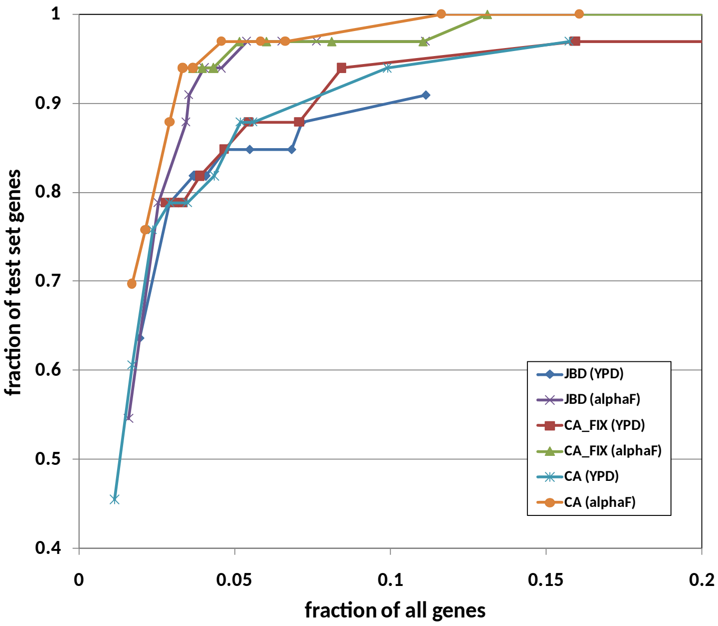

fraction of test set genes
fraction of all genes
1
0.9
0.8
0.7
0.6
0.5
0.4
0
0.05
0.1
0.15
0.2
JBD (YPD)
JBD (alphaF)
CA_FIX (YPD)
CA_FIX (alphaF)
CA (YPD)
CA (alphaF)
