# Supplementary material for: The Evolution of Combinatorial Gene Regulation in Fungi
Source: PLoS Biol. 2008 Feb 26;6(2):e38. doi: 10.1371/journal.pbio.0060038 (PMC2253631; doi:10.1371/journal.pbio.0060038)
Supplement: Supplementary file 8 [file pbio.0060038.sg006.doc]

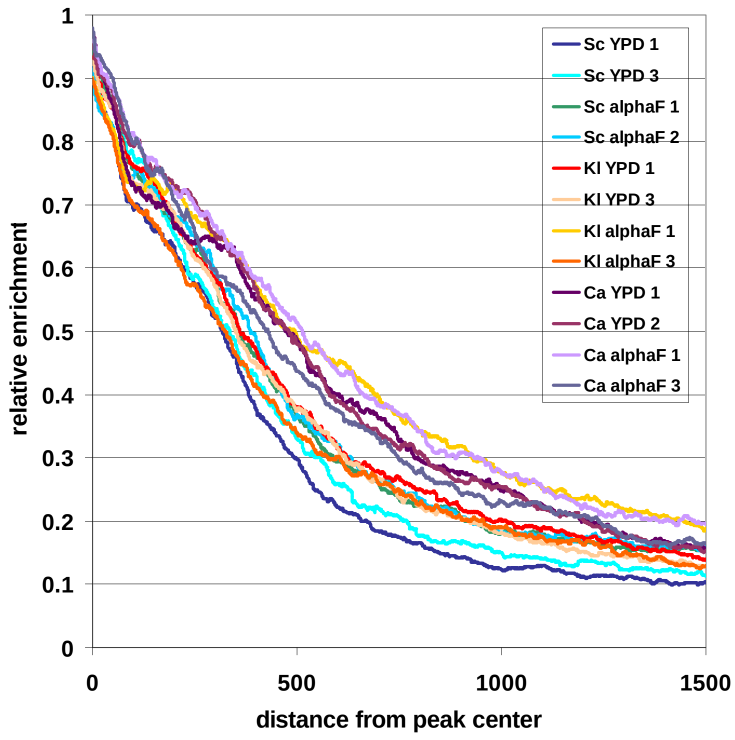
1
0.9
0.8
0.7
0.6
0.5
0.4
0.3
0.2
0.1
0
relative enrichment
0
500
1000
1500
distance from peak center
Sc YPD 1
Sc YPD 3
Sc alphaF 1
Sc alphaF 2
Kl YPD 1
Kl YPD 3
Kl alphaF 1
Kl alphaF 3
Ca YPD 1
Ca YPD 2
Ca alphaF 1
Ca alphaF 3
