# Supplementary material for: The Evolution of Combinatorial Gene Regulation in Fungi
Source: PLoS Biol. 2008 Feb 26;6(2):e38. doi: 10.1371/journal.pbio.0060038 (PMC2253631; doi:10.1371/journal.pbio.0060038)
Supplement: Supplementary file 9 [file pbio.0060038.sg009.doc]

Species B

Number of genes bound in species A mapped to species B

Species A	S. cerevisiae	K. lactis	C. albicans	Total genes bound
**S. cerevisiae**		111	92	247
**K. lactis**	291		349	626
**C. albicans**	268	305		761

Fraction of genes mapped from species A bound in species B

Species A	S. cerevisiae	K. lactis	C. albicans
**S. cerevisiae**		42%	22%
**K. lactis**	16%		19%
**C. albicans**	7%	22%	

Species A	S. cerevisiae	K. lactis	C. albicans
**S. cerevisiae**		22.1	3.8
**K. lactis**	22.1		8.4
**C. albicans**	3.8	8.4	

motif p-value	0.1
$p_{binding}$	0.2
$\sum(p_{binding} * strength_{binding})$	0.5
$\sum(p_{binding} * strength_{binding})$ override	2.0
ortholog mapping	1:1

Species B

Number of genes bound in species A mapped to species B

Species A	S. cerevisiae	K. lactis	C. albicans	Total genes bound
**S. cerevisiae**		99	84	224
**K. lactis**	255		303	549
**C. albicans**	233	267		655

Fraction of genes mapped from species A bound in species B

Species A	S. cerevisiae	K. lactis	C. albicans
**S. cerevisiae**		39%	15%
**K. lactis**	15%		17%
**C. albicans**	6%	19%	

Species A	S. cerevisiae	K. lactis	C. albicans
**S. cerevisiae**		19.0	1.9
**K. lactis**	19.0		6.7
**C. albicans**	1.9	6.7	

motif p-value	0.01
$p_{binding}$	0.2
$\sum(p_{binding} * strength_{binding})$	0.5
$\sum(p_{binding} * strength_{binding})$ override	2.0
ortholog mapping	1:1

Species B

Number of genes bound in species A mapped to species B

Species A	S. cerevisiae	K. lactis	C. albicans	Total genes bound
**S. cerevisiae**		119	98	260
**K. lactis**	326		384	720
**C. albicans**	303	349		884

Fraction of genes mapped from species A bound in species B

Species A	S. cerevisiae	K. lactis	C. albicans
**S. cerevisiae**		44%	22%
**K. lactis**	16%		19%
**C. albicans**	7%	21%	

Species A	S. cerevisiae	K. lactis	C. albicans
**S. cerevisiae**		22.8	3.6
**K. lactis**	22.8		7.1
**C. albicans**	3.6	7.1	

motif p-value	0.1
$p_{binding}$	0.2
$\sum(p_{binding} * strength_{binding})$	0.5
$\sum(p_{binding} * strength_{binding})$ override	1.0
ortholog mapping	1:1

Species B

Number of genes bound in species A mapped to species B

Species A	S. cerevisiae	K. lactis	C. albicans	Total genes bound
**S. cerevisiae**		176	141	247
**K. lactis**	439		425	626
**C. albicans**	373	397		761

Fraction of genes mapped from species A bound in species B

Species A	S. cerevisiae	K. lactis	C. albicans
**S. cerevisiae**		43%	25%
**K. lactis**	15%		21%
**C. albicans**	9%	23%	

Species A	S. cerevisiae	K. lactis	C. albicans
**S. cerevisiae**		31.3	7.4
**K. lactis**	21.5		10.7
**C. albicans**	5.5	11.0	

motif p-value	0.1
$p_{binding}$	0.2
$\sum(p_{binding} * strength_{binding})$	0.5
$\sum(p_{binding} * strength_{binding})$ override	2.0
ortholog mapping	2:1

Species B

Number of genes bound in species A mapped to species B

Species A	S. cerevisiae	K. lactis	C. albicans	Total genes bound
**S. cerevisiae**		112	90	256
**K. lactis**	263		313	560
**C. albicans**	250	289		729

Fraction of genes mapped from species A bound in species B

Species A	S. cerevisiae	K. lactis	C. albicans
**S. cerevisiae**		38%	17%
**K. lactis**	16%		17%
**C. albicans**	6%	18%	

Species A	S. cerevisiae	K. lactis	C. albicans
**S. cerevisiae**		20.0	2.1
**K. lactis**	20.0		5.5
**C. albicans**	2.1	5.5	

motif p-value	0.1
$p_{binding}$	0.05
$\sum(p_{binding} * strength_{binding})$	0.5
$\sum(p_{binding} * strength_{binding})$ override	∞
ortholog mapping	1:1

Species B

Number of genes bound in species A mapped to species B

Species A	S. cerevisiae	K. lactis	C. albicans	Total genes bound
**S. cerevisiae**		107	88	242
**K. lactis**	252		305	538
**C. albicans**	239	273		682

Fraction of genes mapped from species A bound in species B

Species A	S. cerevisiae	K. lactis	C. albicans
**S. cerevisiae**		40%	17%
**K. lactis**	17%		16%
**C. albicans**	6%	18%	

Species A	S. cerevisiae	K. lactis	C. albicans
**S. cerevisiae**		21.7	2.4
**K. lactis**	21.7		5.5
**C. albicans**	2.4	5.5	

motif p-value	0.1
$p_{binding}$	0.2
$\sum(p_{binding} * strength_{binding})$	0.5
$\sum(p_{binding} * strength_{binding})$ override	∞
ortholog mapping	1:1
